# Double salt crystal structure of nona­sodium dihydrogen nona­vanadoplatinate(IV) tri­hydrogen nona­vanadoplatinate(IV) tetra­contahydrate: stepwise-protonated nona­vanadoplatinate(IV)

**DOI:** 10.1107/S2056989015010956

**Published:** 2015-06-13

**Authors:** Hea-Chung Joo, Ki-Min Park, Uk Lee

**Affiliations:** a Department of Chemistry, Pukyong National University, 599-1 Daeyeon 3-dong, Nam-gu, Busan 608-737, Republic of Korea; b Research Institute of Natural Science, Gyeongsan National University, 501, Jinju-daero, Jinju, 660-701, Republic of Korea

**Keywords:** crystal structure, protonated nona­vanadoplatinate(IV), heteropolyoxovanadate containing platinum(IV), hydrogen bonding

## Abstract

Nonavanadoplatinate [Pt^IV^V_9_O_28_]^7−^, which is the first heteropolyoxovanadate in the deca­vanadate framework, [V_10_O_28_]^6−^, has been investigated crystallographically. The title compound, Na_9_[H_2_Pt^IV^V_9_O_28_][H_3_Pt^IV^V_9_O_28_]·40H_2_O, was obtained by a hydro­thermal reaction at pH = 2. This compound contains two different protonated heteropolyoxovanadates, [H_2_Pt^IV^V_9_O_28_]^5−^ [polyanion (*A*)] and [H_3_Pt^IV^V_9_O_28_]^7−^ [polyanion (*B*)]. The locations of the H atoms on the protonated O atoms were observed in difference Fourier maps and confirmed by the inter­polyanion hydrogen bonds, bond-length elongation and bond-valence-sum (VBS) analysis. The two (Pt and V)-bound μ_2_-O atoms are protonated in both polyanions. The position of the third protonated O atom in polyanion (*B*) is an inter­esting feature of the structure, being located on one (V_2_)-bound μ_2_-O atom. The discrete heteropolyanions form a dimer, {H_5_[PtV_9_O_28_]_2_}^9−^, through five inter­polyanion hydrogen bonds. Additional O—H⋯O hydrogen bonds and interactions between Na^+^ cations and water molecules as well as terminal O atoms of one of the polyanions consolidate the crystal packing.

## Abstract 2

Nonavanadoplatinate [Pt^IV^V_9_O_28_]^7−^, which is the first heteropolyoxovanadate in the deca­vanadate framework, [V_10_O_28_]^6−^, has been investigated crystallographically. The title compound, Na_9_[H_2_Pt^IV^V_9_O_28_][H_3_Pt^IV^V_9_O_28_]·40H_2_O, was obtained by a hydro­thermal reaction at pH = 2 and contains two different protonated heteropolyoxovanadates.

## Chemical context   

The deca­vanadate, [V_10_O_28_]^6−^ (Evans, 1966 ▸), is a common isopolyanion in vanadium systems, although no corresponding framework species have been observed in Mo and W systems. The deca­vanadate framework has been substituted with Pt^IV^, and we have previously reported structural studies and ^195^Pt and ^51^V NMR studies of the sodium salt, Na_5_[H_2_PtV_9_O_28_]·21H_2_O (Lee *et al.*, 2008 ▸). The same heteropolyanions were also reported as the guanidinium salt, (CH_6_N_3_)_5_[H_2_PtV_9_O_28_] (Joo *et al.*, 2011 ▸) and as the potassium salt, K_5_[H_2_PtV_9_O_28_]·9H_2_O (Joo & Lee, 2015 ▸).

Konaka *et al.* (2011 ▸) reported heteropolyanions that belong to the deca­vanadate structure type, including the Te derivative, [H_*n*_TeV_9_O_28_]^(5−*n*)−^ (*n* = 1 or 2). However, the Te heteroatom in that polyanion was disordered over two sites, which correspond to the Pt1 or Pt2 and V4 or V13 sites in the title compound. In contrast, the Pt atom shows no disorder in any of the three [H_2_PtV_9_O_28_]^5−^ polyanions reported thus far. Recently, the crystal structures of Fe- and Ni-substituted deca­niobates, [H_2_Fe^III^Nb_9_O_28_]^6−^ and [H_3_Ni^II^Nb_9_O_28_]^6−^ were reported as tetra­methyl­ammonium salts (Son *et al.*, 2013 ▸).

In our studies of Anderson-type heteropolyoxometalates (Anderson, 1937 ▸) containing Pt^IV^, [PtH*_n_M*_6_O_24_]^(8−*n*)−^ (*M =* Mo or W), we have found that gradual protonation is a typical characteristic of these compounds (Lee & Joo, 2004 ▸; Izarova *et al.*, 2012 ▸; Joo *et al.*, 2015 ▸). We herein report the crystal structure of the title compound, a double salt containing stepwise-protonated nona­vanadoplatinate(IV) by two and three H^+^ ions.

## Structural commentary   

**Figure 1:**
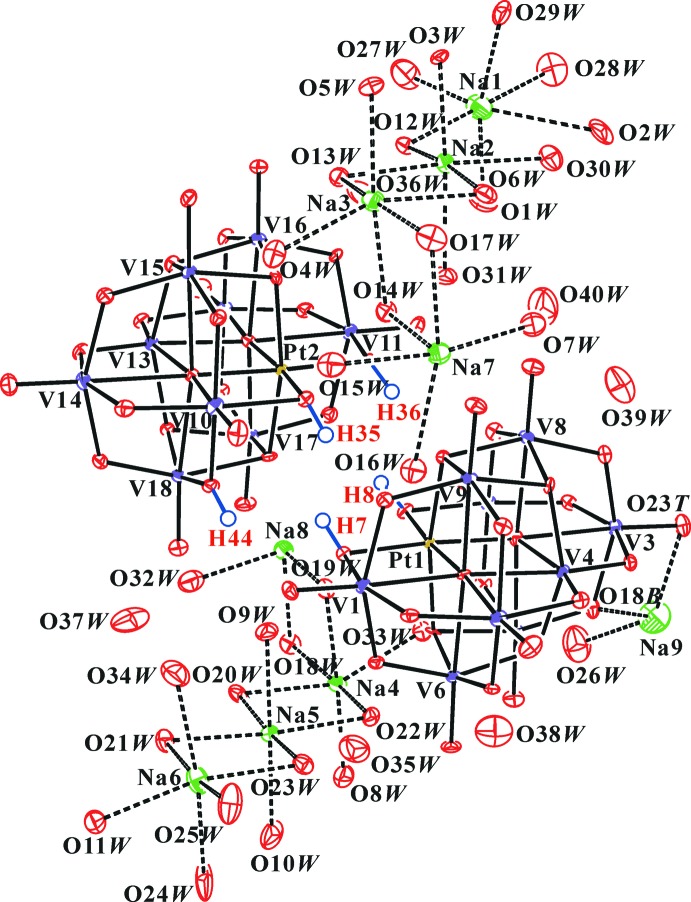
The mol­ecular entities in the crystal structure of the title compound. Displacement ellipsoids are drawn at the 30% probability level. The H atoms of the polyanion are presented as small spheres of arbitrary radius and the H atoms of water mol­ecules have been omitted for clarity.

**Figure 2:**
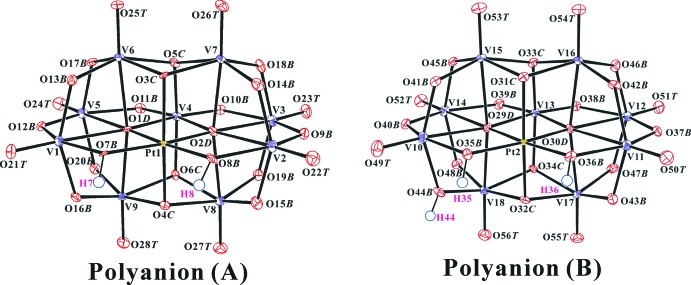
The polyanion structure in the title compound with the atomic numbering scheme and displacement ellipsoids at the 30% probability level for non-H atoms. H atoms are presented as a small spheres of arbitrary radius.

The title compound contains doubly and triply protonated nona­vanadoplatinates, [H_2_Pt^IV^V_9_O_28_]^5−^ [polyanion (*A*)] and [H_3_Pt^IV^V_9_O_28_]^4−^ [polyanion (*B*)]. Fig. 1 ▸ shows the structure of the title compound while Fig. 2 ▸ shows the structure of polyanions (*A*) and (*B*). The framework of [V_10_O_28_]^6−^ has been studied in detail previously (Evans, 1966 ▸; Nowogrocki *et al.*, 1997 ▸). The O atoms of the polyanions were designated as O*T* (terminal V=O atom), O*B* (bridging μ_2_-O atom; V—O—V or V—O—Pt), O*C* {μ_3_-O atom; (V)_3_—O or (V)_2_—O—Pt} and O*D* {μ_4_-O atom; (V)_4_—O or (V)_3_—O—PT}. The nine [VO_6_] octa­hedra in the polyanions are distorted [V—O ranges 1.578 (6)–2.419 (5)Å], whereas in the [PtO_6_] octa­hedron, the Pt—O distances are all very similar [Pt—O ranges 1.966 (5)–2.025 (5) Å]. The H atoms of the protonated O atoms were found in difference Fourier maps and confirmed by the presence of inter­polyanion hydrogen bonds (Fig. 2 ▸), bond-length elongation of V—OH (Table 1 ▸), and the bond-valence sum (BVS; Brown & Altermatt, 1985 ▸; Brese & O’Keeffe, 1991 ▸) analysis.

**Figure 3:**
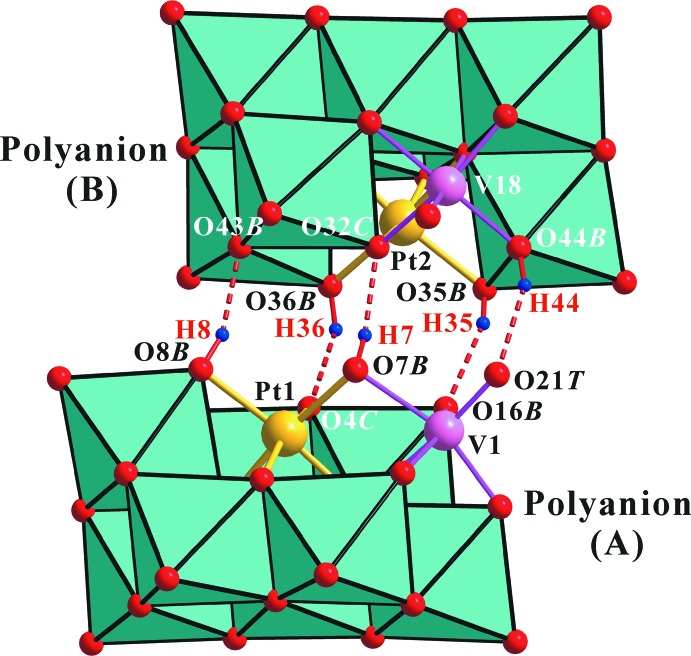
Polyhedral view of the heteropolyanion in the title compound, with O—H⋯O contacts of the inter­anion hydrogen bonds shown as red dashed lines.

The two (Pt and V)-bound *μ*_2_-O atoms, O7*B*, O8*B* in polyanion (*A*) and O35*B*, O36*B* in polyanion (*B*), are proton­ated. In addition, one (V_2_)-bound *μ*_2_-O, O44*B* in polyanion (*B*), is also protonated. These protons are particularly important in the solid state. Polyanion *A* and *B* are involved in forming a dimeric assembly, {H_5_[PtV_9_O_28_]_2_}^9−^, which is held together by two (Pt and V)-bound *μ*_2_-O(8*B* and 35*B*)–H(8 and 35)⋯(V_2_)-bound *μ*_2_-O(43*B* and 16*B*) (bridged O atom), two (Pt and V)-bound *μ*_2_-O(7*B* and 36*B*)— H(7 and 36)⋯(Pt and V_2_)-bound *μ*_3_-O(4*C* and 32*C*), and one (V_2_)-bound *μ*_2_-O44*B*—H44⋯ *μ*_1_-O21*T* (terminal O atom) hydrogen bonds (Fig. 3 ▸ and Table 2 ▸), respectively. The O44*B*⋯O21*T* distance of 2.724 (8) Å is shorter than that of O15*B*⋯O50*T* [2.889 (8) Å] because O44*B*—H44⋯O21*T* forms hydrogen bonds. Considering the bond-length elongation of V10—O44*B* and V18—O44*B* in polyanion (*B*) and the bond angles of V10—O44*B*—V18, the O44*B* atoms should be protonated by H44 in polyanion (*B*) (Table 1 ▸).

Confirmation of the protonated O atoms was strongly supported by the BVS analysis. The BVSs for O7*B* and O8*B* in polyanion (*A*), and for O35*B*, O36*B*, and O44*B* in polyanion (*B*) are 1.20, 1.15, 1.19, 1.22, and 1.42 valence units (v.u.), respectively, if the valence of the O—H bond is not included. Because the BVS value around the *μ*_2_-O (O*B*) atom should be 2.0 v.u., the missing valences of O7*B*, O8*B*, O35*B*, O36*B*, and O44*B* are 0.80, 0.85, 0.81, 0.78, and 0.58 v.u., respectively, corresponding to the valence of the O—H bonds. The BVSs around the other unprotonated *μ*_2_-O atoms in polyanion (*B*) for O41*B–*-O43*B* and O45*B*—O48*B* are 1.82, 1.80, 1.71, 1.81, 1.79, 1.79, and 1.83 v.u., respectively, if the valence of the O*B*⋯H—O*W* hydrogen bonds and O*B*⋯Na^+^ inter­actions are not included. The missing valences of these O*B* atoms correspond to the valences of the O*B*⋯H—O*W* hydrogen bonds and O*B*⋯Na^+^ inter­actions. The smallest BVS value in the other unprotonated *μ*2-O*B* and *μ*3-O*C* atoms in polyanion (*A*) is 1.69 v.u. for O16*B*. O16*B* in polyanion (*A*) corresponds to O44*B* in polyanion (*B*), which is protonated. Similar results were observed for the sodium (Lee *et al.*, 2008 ▸), guanidinium (Joo *et al.*, 2011 ▸), and potassium (Joo & Lee, 2015 ▸) salts of [H_2_PtV_9_O_28_]^5−^.

The Na1–Na6 ions are coordinated by six O*W* atoms in the range 2.339 (8)–2.742 (9) Å, and the Na7 and Na8 ions are coord­in­ated by five O*W* and one O*T* atoms in the range 2.373 (6)–2.454 (6) Å. The Na9 ions are coordinated by four O*W* atoms in the range 2.204 (11)–2.410 (9) Å.

## Supra­molecular features   

Polyanions (*A*) and (*B*) are involved in forming the dimeric assembly, {H_5_[PtV_9_O_28_]_2_}^9−^. Furthermore, the polyanion dimers are three-dimensionally linked *via* Na^+^⋯O*T* inter­actions. All water mol­ecules except O15*W*, O26*W*, O27*W*, O30*W* and O32*W* are involved in an extensive hydrogen-bonding network with O atoms of the polyanions. (see Table 2 ▸). Potential hydrogen-bond distances of O35*W*–O40*W* molecules are: O35*W*⋯O8*W* ^iv^ 2.845 (10); O35*W*⋯O38*W* 2.735 (12); O36*W*⋯O1*W* 2.814 (11); O36*W*⋯O5*W* ^viii^ 2.846 (11); O37*W*⋯O27*T* ^vi^ 2.844 (9); O37*W*⋯O55*T* 2.952 (9); O38*W*⋯O24*T* ^viii^ 2.828 (10); O39*W*⋯O40*W* 2.793 (14); O40*W*⋯O1*W* 2.778 (13); O40*W*⋯O50*T* 2.913 (11) Å (symmetry codes correspond to those in Table 2 ▸).

## Database survey   

A number of nona­vanadoplatinate(IV) compounds have been reported: Na_5_[H_2_PtV_9_O_28_]·21H_2_O (Lee *et al.*, 2008 ▸); (CH_6_N_3_)_5_[H_2_PtV_9_O_28_] (Joo *et al.*, 2011 ▸); K_5_[H_2_PtV_9_O_28_]·9H_2_O (Joo & Lee, 2015 ▸). In addition, related structures of nona­vanadotellurate(VI), and nona­niobatoferrate(III) and nona­niobatonickelate(II) have been reported: [H_*n*_TeV_9_O_28_]^(5−*n*)−^ (*n* = 1 or 2) (Konaka *et al.*, 2011 ▸); [H_2_Fe^III^Nb_9_O_28_]^6−^ and [H_3_Ni^II^Nb_9_O_28_]^6−^ (Son *et al.*, 2013 ▸).

## Synthesis and crystallization   

Single crystals of the title compound were obtained in the same way as the sodium salt reported by Lee *et al.* (2008 ▸), at pH 2.0.

## Refinement   

Crystal data, data collection and structure refinement details are summarized in Table 3 ▸. All H atoms in the polyanions and water mol­ecules O1*W*–O11*W* were found in difference Fourier maps, and were refined with 1,2 and 1,3 distance restraints of O—H = 0.85 (3) Å and H⋯H = 1.50 (2) Å, respectively using the command DFIX in *SHELXL2014/7* (Sheldrick, 2015 ▸) and were included in the refinement with *U*_iso_(H) = 1.5 *U*_eq_(O). The H atoms of O12*W*–O26*W* were positioned geometrically and refined using a riding model (HFIX 23), with O*W*—H = 0.99 Å and *U*_iso_ (H) = 1.5*U*_eq_ (O). The H atoms of O27*W*–O34*W* were positioned geometrically and refined using a riding model (HFIX 137), with O*W*—H = 0.98 Å and *U*_iso_ (H) = 1.5*U*_eq_ (O). All invalid H atoms were removed in the final step of refinement. The H atoms of O35*W*–O40*W* were omitted in the refinement because they were not coordinated to Na^+^ ions and because they generated level A alerts in the *checkCIF* program due to short inter­molecular D—H⋯H—D contacts. The highest peak in the difference map was 1.78 Å from H17*B* and the largest hole is 0.87 Å from Pt2. The highest peak was considered as a half-occupancy water mol­ecule but it was excluded in the final stage of refinement because it was too close to the neighboring water mol­ecule.

## Supplementary Material

Crystal structure: contains datablock(s) New_Global_Publ_Block, I. DOI: 10.1107/S2056989015010956/pk2554sup1.cif

Structure factors: contains datablock(s) I. DOI: 10.1107/S2056989015010956/pk2554Isup2.hkl

CCDC reference: 1405348

Additional supporting information:  crystallographic information; 3D view; checkCIF report

## Figures and Tables

**Table 1:** Selected bond lengths ()

Pt1O7*B*	2.012(5)	Pt2O35*B*	2.015(5)
Pt1O8*B*	2.025(5)	Pt2O36*B*	2.017(5)
V1O12*B*	1.830(5)	V10O41*B*	1.820(5)
V1O16*B*	1.882(5)	V10O44*B*	1.950(6)
V1O13*B*	1.882(5)	V10O35*B*	2.059(5)
V1O7*B*	2.058(5)	V11O42*B*	1.882(5)
V2O14*B*	1.841(6)	V11O43*B*	1.905(6)
V2O15*B*	1.904(5)	V11O36*B*	2.035(5)
V2O8*B*	2.076(5)	V12O46*B*	1.853(5)
V3O19*B*	1.840(5)	V12O47*B*	1.869(6)
V3O18*B*	1.883(6)	V12O38*B*	2.103(5)
V3O10*B*	2.054(5)	V13O38*B*	1.666(5)
V4O11*B*	1.675(5)	V13O39*B*	1.683(5)
V5O17*B*	1.838(5)	V14O45*B*	1.854(5)
V5O12*B*	1.842(6)	V14O48*B*	1.904(5)
V5O20*B*	1.896(5)	V14O39*B*	2.048(5)
V5O11*B*	2.059(5)	V15O45*B*	1.824(5)
V6O13*B*	1.805(5)	V15O41*B*	1.859(5)
V6O17*B*	1.843(5)	V16O42*B*	1.808(5)
V7O18*B*	1.802(5)	V16O46*B*	1.834(5)
V7O14*B*	1.847(5)	V17O47*B*	1.821(5)
V8O15*B*	1.808(5)	V17O43*B*	1.822(6)
V8O19*B*	1.840(5)	V18O48*B*	1.779(5)
V9O20*B*	1.805(5)	V18O44*B*	1.907(5)
V9O16*B*	1.851(5)		

**Table 2:** Hydrogen-bond geometry (, )

*D*H*A*	*D*H	H*A*	*D* *A*	*D*H*A*
O7*B*H7O32*C*	0.85(3)	1.79(3)	2.627(7)	171(8)
O8*B*H8O43*B*	0.86(3)	1.89(3)	2.737(7)	169(8)
O35*B*H35O16*B*	0.85(3)	1.86(4)	2.685(7)	164(9)
O36*B*H36O4*C*	0.84(3)	1.79(3)	2.628(7)	174(8)
O44*B*H44O21*T*	0.84(3)	2.00(6)	2.724(8)	144(8)
O1*W*H1*B*O40*B* ^i^	0.90(3)	2.18(9)	2.845(9)	130(9)
O2*W*H2*B*O47*B* ^ii^	0.88(3)	1.88(3)	2.757(8)	178(12)
O3*W*H3*A*O39*B* ^ii^	0.86(3)	2.01(4)	2.856(9)	168(10)
O3*W*H3*B*O33*C* ^iii^	0.87(3)	1.97(6)	2.795(8)	159(12)
O4*W*H4*A*O51*T* ^iv^	0.84(3)	2.08(3)	2.893(8)	163(8)
O4*W*H4*B*O41*B*	0.84(3)	1.96(4)	2.780(8)	165(10)
O5*W*H5*B*O46*B* ^iii^	0.86(3)	1.94(5)	2.743(8)	154(9)
O6*W*H6*A*O7*W*	0.85(3)	2.03(3)	2.872(9)	170(9)
O6*W*H6*B*O48*B* ^ii^	0.84(3)	2.02(5)	2.810(8)	158(9)
O7*W*H7*A*O28*T*	0.83(3)	2.26(5)	2.951(8)	141(8)
O7*W*H7*B*O34*W* ^ii^	0.84(3)	2.05(6)	2.794(10)	148(8)
O8*W*H8*A*O17*B* ^v^	0.86(3)	1.93(5)	2.760(8)	161(9)
O8*W*H8*B*O23*T* ^vi^	0.85(3)	2.12(7)	2.872(9)	148(10)
O9*W*H9*A*O7*B*	0.85(3)	2.01(5)	2.826(8)	161(9)
O9*W*H9*B*O37*W*	0.84(3)	2.39(6)	3.073(11)	138(8)
O10*W*H10*A*O5*C* ^v^	0.88(3)	1.92(4)	2.782(9)	165(11)
O11*W*H11*A*O22*T* ^vii^	0.87(3)	2.23(5)	3.061(8)	158(9)
O12*W*H12*A*O42*B*	0.99	1.92	2.861(8)	159
O12*W*H12*B*O53*T* ^iii^	0.99	2.00	2.956(8)	160
O13*W*H13*A*O31*C*	0.99	1.84	2.831(7)	176
O13*W*H13*B*O54*T* ^iii^	0.99	1.93	2.905(8)	169
O14*W*H14*A*O35*B*	0.99	1.96	2.806(8)	142
O14*W*H14*B*O31*W*	0.99	2.03	2.928(8)	151
O15*W*H15*A*O4*W*	0.99	1.92	2.819(9)	149
O16*W*H16*A*O20*B*	0.99	2.51	3.054(7)	115
O16*W*H16*B*O33*W* ^iv^	0.99	1.88	2.764(9)	147
O17*W*H17*A*O37*B* ^iv^	0.99	2.06	3.013(8)	162
O17*W*H17*A*O50*T* ^iv^	0.99	2.60	3.205(8)	119
O18*W*H18*A*O39*W* ^vi^	0.99	1.97	2.942(10)	167
O18*W*H18*B*O12*B* ^viii^	0.99	2.01	2.977(8)	165
O19*W*H19*A*O9*W*	0.99	2.05	2.949(9)	149
O19*W*H19*B*O8*B*	0.99	1.99	2.867(8)	146
O20*W*H20*A*O32*W*	0.99	1.94	2.857(9)	152
O20*W*H20*B*O19*B* ^vi^	0.99	1.81	2.753(8)	157
O21*W*H21*A*O37*W*	0.99	1.93	2.836(10)	151
O21*W*H21*B*O6*C* ^vi^	0.99	1.90	2.869(8)	164
O22*W*H22*A*O25*T* ^v^	0.99	1.94	2.920(8)	170
O22*W*H22*B*O3*C*	0.99	1.83	2.819(7)	173
O23*W*H23*A*O26*T* ^v^	0.99	2.04	2.993(8)	160
O23*W*H23*B*O13*B*	0.99	1.87	2.840(8)	167
O24*W*H24*A*O11*B* ^vi^	0.99	1.97	2.887(9)	152
O24*W*H24*B*O10*W*	0.99	2.15	3.082(13)	157
O25*W*H25*A*O9*B* ^vii^	0.99	2.07	2.874(9)	137
O25*W*H25*B*O35*W*	0.99	1.93	2.880(10)	160
O26*W*H26*A*O11*W* ^i^	0.99	1.88	2.848(11)	165
O26*W*H26*B*O38*W*	0.99	1.86	2.811(12)	161
O27*W*H27*A*O36*W*	0.98	2.11	2.881(10)	135
O27*W*H27*B*O29*W* ^ix^	0.98	2.09	2.891(10)	138
O28*W*H28*A*O27*W* ^ix^	0.98	1.78	2.692(11)	153
O28*W*H28*B*O40*B* ^i^	0.98	2.45	2.994(9)	115
O29*W*H29*A*O38*B* ^ii^	0.98	2.12	2.992(8)	147
O29*W*H29*B*O45*B* ^iii^	0.98	1.93	2.755(8)	141
O30*W*H30*A*O2*W*	0.98	2.03	2.877(10)	143
O30*W*H30*B*O37*W* ^ii^	0.98	1.93	2.893(10)	167
O31*W*H31*B*O36*B*	0.98	2.08	2.812(8)	130
O32*W*H32*A*O40*W* ^vi^	0.98	1.96	2.901(13)	160
O32*W*H32*B*O2*W* ^vi^	0.98	2.01	2.896(10)	150
O33*W*H33*A*O38*W*	0.98	2.13	2.833(11)	128
O33*W*H33*B*O14*B*	0.98	1.81	2.786(8)	178
O34*W*H34*A*O55*T*	0.98	2.29	3.038(9)	132
O34*W*H34*B*O39*W* ^vii^	0.98	2.02	2.979(12)	166

Symmetry codes: (i) 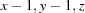; (ii) 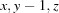; (iii) 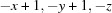; (iv) 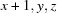; (v) 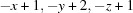; (vi) 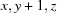; (vii) 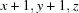; (viii) 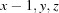; (ix) 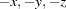.

**Table 3:** Experimental details

Crystal data	
Chemical formula	Na_9_[H_2_PtV_9_O_28_][H_3_PtV_9_O_28_]40H_2_O
*M*_r_	1567.84
Crystal system, space group	Triclinic, *P* 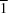
Temperature (K)	173
*a*, *b*, *c* ()	12.706(1), 12.875(1), 28.319(2)
, , ()	93.760(1), 98.449(1), 113.318(1)
*V* (^3^)	4168.9(5)
*Z*	4
Radiation type	Mo *K*
(mm^1^)	5.44
Crystal size (mm)	0.40 0.20 0.20

Data collection	
Diffractometer	Bruker SMART CCD
Absorption correction	Multi-scan (*SADABS*; Bruker, 1997 ▸)
*T*_min_, *T*_max_	0.515, 0.746
No. of measured, independent and observed [*I* > 2(*I*)] reflections	37312, 19046, 13416
*R*_int_	0.039
(sin /)_max_ (^1^)	0.666

Refinement	
*R*[*F* ^2^ > 2(*F* ^2^)], *wR*(*F* ^2^), *S*	0.053, 0.115, 1.05
No. of reflections	19046
No. of parameters	1203
No. of restraints	38
H-atom treatment	Only H-atom coordinates refined
_max_, _min_ (e ^3^)	2.99, 2.15

Computer programs: *SMART* and *SAINT* (Bruker, 1997 ▸), *SHELXL2014* (Sheldrick, 2015 ▸), *ORTEP-3 for Windows* (Farrugia, 2012 ▸) and *DIAMOND* (Brandenburg, 1998 ▸).

## supplementary crystallographic information

### Crystal data

**Table:**

Na_9_[H_2_PtV_9_O_28_][H_3_PtV_9_O_28_]·40H_2_O	*Z* = 4
*M**_r_* = 1567.84	*F*(000) = 3044
Triclinic, *P*1	*D*_x_ = 2.498 Mg m^−^^3^
*a* = 12.706 (1) Å	Mo *K*α radiation, λ = 0.71073 Å
*b* = 12.875 (1) Å	Cell parameters from 7031 reflections
*c* = 28.319 (2) Å	θ = 2.5–28.3°
α = 93.760 (1)°	µ = 5.44 mm^−^^1^
β = 98.449 (1)°	*T* = 173 K
γ = 113.318 (1)°	Block, dark brown
*V* = 4168.9 (5) Å^3^	0.40 × 0.20 × 0.20 mm

### Data collection

**Table:**

Bruker SMART CCD diffractometer	19046 independent reflections
Radiation source: fine-focus sealed tube	13416 reflections with *I* > 2σ(*I*)
Detector resolution: 10.0 pixels mm^-1^	*R*_int_ = 0.039
φ and ω scans	θ_max_ = 28.3°, θ_min_ = 1.7°
Absorption correction: multi-scan (*SADABS*; Bruker, 1997)	*h* = −16→16
*T*_min_ = 0.515, *T*_max_ = 0.746	*k* = −16→16
37312 measured reflections	*l* = −36→36

### Refinement

**Table:**

Refinement on *F*^2^	Secondary atom site location: difference Fourier map
Least-squares matrix: full	Hydrogen site location: difference Fourier map
*R*[*F*^2^ > 2σ(*F*^2^)] = 0.053	Only H-atom coordinates refined
*wR*(*F*^2^) = 0.115	*w* = 1/[σ^2^(*F*_o_^2^) + (0.0047*P*)^2^ + 66.1402*P*] where *P* = (*F*_o_^2^ + 2*F*_c_^2^)/3
*S* = 1.05	(Δ/σ)_max_ = 0.001
19046 reflections	Δρ_max_ = 2.99 e Å^−^^3^
1203 parameters	Δρ_min_ = −2.15 e Å^−^^3^
38 restraints	Extinction correction: *SHELXL2014* (Sheldrick, 2015), Fc^*^=kFc[1+0.001xFc^2^λ^3^/sin(2θ)]^-1/4^
Primary atom site location: structure-invariant direct methods	Extinction coefficient: 0.00027 (2)

### Special details

**Table:**

Geometry. All esds (except the esd in the dihedral angle between two l.s. planes) are estimated using the full covariance matrix. The cell esds are taken into account individually in the estimation of esds in distances, angles and torsion angles; correlations between esds in cell parameters are only used when they are defined by crystal symmetry. An approximate (isotropic) treatment of cell esds is used for estimating esds involving l.s. planes.

### Fractional atomic coordinates and isotropic or equivalent isotropic displacement parameters (Å^2^)

**Table:**

	*x*	*y*	*z*	*U*_iso_*/*U*_eq_	
Pt1	0.45003 (2)	0.74403 (2)	0.33507 (2)	0.00550 (7)	
V1	0.71159 (11)	0.91128 (11)	0.33797 (5)	0.0103 (3)	
V2	0.18401 (11)	0.58053 (11)	0.33073 (5)	0.0110 (3)	
V3	0.26993 (11)	0.45141 (12)	0.40609 (5)	0.0121 (3)	
V4	0.53061 (10)	0.61272 (11)	0.41324 (5)	0.0085 (3)	
V5	0.78830 (11)	0.78352 (11)	0.41622 (5)	0.0114 (3)	
V6	0.60439 (11)	0.88428 (11)	0.43233 (5)	0.0092 (3)	
V7	0.34669 (11)	0.71757 (11)	0.42932 (5)	0.0097 (3)	
V8	0.36930 (11)	0.48243 (11)	0.31023 (5)	0.0107 (3)	
V9	0.63138 (10)	0.64442 (11)	0.31612 (5)	0.0092 (3)	
O1D	0.5982 (4)	0.7545 (4)	0.37248 (18)	0.0073 (10)	
O2D	0.3762 (4)	0.6130 (4)	0.36978 (19)	0.0097 (11)	
O3C	0.4450 (4)	0.8409 (4)	0.39323 (18)	0.0077 (10)	
O4C	0.4671 (4)	0.6306 (4)	0.28785 (18)	0.0090 (11)	
O5C	0.5095 (4)	0.7303 (4)	0.45092 (18)	0.0079 (10)	
O6C	0.5283 (4)	0.5341 (4)	0.35301 (18)	0.0085 (11)	
O7B	0.5441 (4)	0.8818 (4)	0.30612 (19)	0.0097 (11)*	
H7	0.539 (7)	0.871 (7)	0.2760 (12)	0.015*	
O8B	0.2886 (4)	0.7184 (5)	0.30061 (19)	0.0099 (11)	
H8	0.300 (7)	0.719 (7)	0.2716 (15)	0.015*	
O9B	0.1471 (4)	0.4637 (4)	0.36745 (19)	0.0120 (11)	
O10B	0.4427 (4)	0.5006 (4)	0.43593 (19)	0.0107 (11)	
O11B	0.6689 (4)	0.6432 (4)	0.43874 (19)	0.0108 (11)	
O12B	0.8313 (4)	0.8977 (4)	0.37779 (19)	0.0113 (11)	
O13B	0.6724 (4)	0.9768 (4)	0.39029 (19)	0.0110 (11)	
O14B	0.2173 (4)	0.6896 (5)	0.38265 (19)	0.0126 (12)	
O15B	0.2350 (4)	0.4964 (5)	0.2887 (2)	0.0133 (12)	
O16B	0.6928 (4)	0.7852 (4)	0.29471 (19)	0.0119 (11)	
O17B	0.7368 (4)	0.8638 (4)	0.45617 (18)	0.0104 (11)	
O18B	0.2870 (4)	0.5785 (5)	0.4484 (2)	0.0138 (12)	
O19B	0.3080 (4)	0.3884 (4)	0.35454 (19)	0.0125 (11)	
O20B	0.7577 (4)	0.6737 (4)	0.36218 (19)	0.0114 (11)	
O21T	0.7782 (4)	1.0172 (5)	0.3111 (2)	0.0138 (12)	
O22T	0.0600 (5)	0.5678 (5)	0.3020 (2)	0.0159 (12)	
O23T	0.2087 (5)	0.3473 (5)	0.4344 (2)	0.0182 (13)	
O24T	0.9056 (5)	0.7901 (5)	0.4468 (2)	0.0197 (13)	
O25T	0.5988 (5)	0.9681 (5)	0.47535 (19)	0.0147 (12)	
O26T	0.3380 (5)	0.7994 (5)	0.4727 (2)	0.0155 (12)	
O27T	0.3765 (5)	0.3963 (5)	0.2688 (2)	0.0173 (12)	
O28T	0.6421 (5)	0.5584 (5)	0.2754 (2)	0.0153 (12)	
Pt2	0.53911 (2)	0.75266 (2)	0.16508 (2)	0.00586 (7)	
V10	0.80443 (11)	0.91131 (12)	0.17061 (5)	0.0122 (3)	
V11	0.27684 (11)	0.58186 (11)	0.16058 (5)	0.0115 (3)	
V12	0.20221 (11)	0.71474 (11)	0.08364 (5)	0.0114 (3)	
V13	0.46057 (11)	0.88413 (11)	0.08640 (5)	0.0086 (3)	
V14	0.72497 (11)	1.04265 (11)	0.09456 (5)	0.0119 (3)	
V15	0.64396 (11)	0.77619 (11)	0.07139 (5)	0.0097 (3)	
V16	0.38604 (11)	0.61354 (11)	0.06679 (5)	0.0098 (3)	
V17	0.35747 (11)	0.84849 (11)	0.18413 (5)	0.0102 (3)	
V18	0.61454 (11)	1.01700 (11)	0.18801 (5)	0.0102 (3)	
O29D	0.6175 (4)	0.8848 (4)	0.13261 (18)	0.0088 (11)	
O30D	0.3927 (4)	0.7441 (4)	0.12588 (19)	0.0087 (11)	
O31C	0.5470 (4)	0.6575 (4)	0.10733 (18)	0.0086 (11)	
O32C	0.5210 (4)	0.8660 (4)	0.21187 (18)	0.0095 (11)	
O33C	0.4847 (4)	0.7683 (4)	0.04911 (19)	0.0109 (11)	
O34C	0.4619 (4)	0.9637 (4)	0.14678 (18)	0.0096 (11)	
O35B	0.6982 (4)	0.7753 (5)	0.20013 (19)	0.0102 (11)	
H35	0.708 (7)	0.790 (7)	0.2306 (11)	0.015*	
O36B	0.4423 (4)	0.6146 (4)	0.19353 (18)	0.0095 (11)	
H36	0.456 (7)	0.622 (7)	0.2239 (11)	0.014*	
O37B	0.1590 (4)	0.5998 (5)	0.12020 (19)	0.0126 (11)	
O38B	0.3245 (4)	0.8564 (5)	0.05981 (18)	0.0113 (11)	
O39B	0.5532 (4)	0.9973 (5)	0.06518 (19)	0.0121 (11)	
O40B	0.8440 (4)	1.0311 (5)	0.13493 (19)	0.0132 (12)	
O41B	0.7744 (4)	0.8063 (4)	0.11859 (18)	0.0107 (11)	
O42B	0.3181 (4)	0.5186 (4)	0.10828 (18)	0.0108 (11)	
O43B	0.2940 (4)	0.7090 (5)	0.20421 (19)	0.0129 (11)	
O44B	0.7532 (4)	0.9993 (5)	0.21330 (19)	0.0119 (11)	
H44	0.792 (7)	1.017 (7)	0.2416 (15)	0.018*	
O45B	0.7080 (4)	0.9175 (4)	0.05283 (19)	0.0116 (11)	
O46B	0.2550 (4)	0.6353 (5)	0.04306 (19)	0.0125 (11)	
O47B	0.2332 (4)	0.8236 (5)	0.13672 (19)	0.0132 (12)	
O48B	0.6835 (4)	1.1086 (4)	0.14703 (19)	0.0121 (11)	
O49T	0.9260 (5)	0.9248 (5)	0.2009 (2)	0.0199 (13)	
O50T	0.2067 (5)	0.4744 (5)	0.1849 (2)	0.0183 (13)	
O51T	0.0834 (5)	0.7074 (5)	0.0525 (2)	0.0178 (13)	
O52T	0.7884 (5)	1.1476 (5)	0.0677 (2)	0.0180 (13)	
O53T	0.6564 (5)	0.6969 (5)	0.0290 (2)	0.0153 (12)	
O54T	0.3952 (5)	0.5322 (5)	0.02373 (19)	0.0143 (12)	
O55T	0.6068 (5)	1.0995 (5)	0.2310 (2)	0.0179 (12)	
O56T	0.3456 (5)	0.9345 (5)	0.2243 (2)	0.0169 (12)	
Na1	0.1461 (3)	0.1283 (3)	0.07263 (15)	0.0339 (9)	
Na2	0.5008 (3)	0.3083 (3)	0.11131 (12)	0.0174 (7)	
Na3	0.7943 (3)	0.4930 (3)	0.11847 (12)	0.0194 (7)	
Na4	0.2007 (3)	1.0129 (3)	0.38164 (12)	0.0167 (7)	
Na5	0.4888 (3)	1.1888 (3)	0.38675 (11)	0.0155 (7)	
Na6	0.7798 (3)	1.3553 (3)	0.38754 (14)	0.0265 (8)	
Na7	0.9239 (3)	0.5954 (3)	0.23834 (12)	0.0199 (7)	
Na8	0.0689 (3)	0.9108 (3)	0.26382 (12)	0.0197 (7)	
Na9	0.1092 (4)	0.4763 (4)	0.48275 (18)	0.0510 (12)	
O1W	0.0600 (6)	0.2091 (6)	0.1273 (3)	0.0383 (17)	
H1A	0.033 (8)	0.165 (9)	0.098 (2)	0.057*	
H1B	−0.008 (5)	0.194 (10)	0.137 (3)	0.057*	
O2W	0.1628 (7)	−0.0001 (6)	0.1445 (3)	0.0374 (18)	
H2A	0.093 (5)	−0.031 (8)	0.125 (4)	0.056*	
H2B	0.184 (8)	−0.057 (6)	0.143 (4)	0.056*	
O3W	0.5461 (8)	0.2114 (6)	0.0491 (2)	0.044 (2)	
H3A	0.540 (11)	0.142 (4)	0.050 (4)	0.065*	
H3B	0.519 (10)	0.215 (9)	0.0197 (18)	0.065*	
O4W	0.9156 (5)	0.6885 (6)	0.1140 (2)	0.0226 (14)	
H4A	0.954 (7)	0.696 (8)	0.092 (3)	0.034*	
H4B	0.866 (7)	0.717 (8)	0.110 (3)	0.034*	
O5W	0.8156 (6)	0.3811 (6)	0.0545 (2)	0.0289 (15)	
H5A	0.879 (6)	0.441 (6)	0.052 (3)	0.043*	
H5B	0.779 (7)	0.355 (8)	0.0253 (16)	0.043*	
O6W	0.6875 (5)	0.3288 (5)	0.1561 (2)	0.0205 (13)	
H6A	0.711 (8)	0.349 (7)	0.1864 (12)	0.031*	
H6B	0.698 (8)	0.270 (5)	0.148 (3)	0.031*	
O7W	0.7774 (6)	0.4251 (6)	0.2556 (2)	0.0247 (14)	
H7A	0.734 (7)	0.440 (8)	0.271 (3)	0.037*	
H7B	0.803 (8)	0.378 (7)	0.266 (3)	0.037*	
O8W	0.1747 (6)	1.1174 (6)	0.4471 (2)	0.0305 (16)	
H8A	0.187 (9)	1.110 (8)	0.4771 (15)	0.046*	
H8B	0.212 (9)	1.186 (4)	0.443 (3)	0.046*	
O9W	0.4860 (6)	1.0706 (6)	0.3198 (2)	0.0271 (15)	
H9A	0.495 (9)	1.009 (5)	0.321 (3)	0.041*	
H9B	0.478 (9)	1.085 (8)	0.2913 (16)	0.041*	
O10W	0.4923 (8)	1.3096 (7)	0.4538 (3)	0.046 (2)	
H10A	0.503 (9)	1.295 (11)	0.4835 (19)	0.069*	
H10B	0.418 (4)	1.294 (12)	0.447 (4)	0.069*	
O11W	0.8894 (6)	1.5425 (5)	0.3708 (2)	0.0250 (14)	
H11A	0.938 (6)	1.568 (8)	0.351 (3)	0.037*	
H11B	0.823 (4)	1.497 (7)	0.351 (3)	0.037*	
O12W	0.3096 (5)	0.3022 (5)	0.0720 (2)	0.0181 (12)	
H12A	0.2971	0.3655	0.0884	0.027*	
H12B	0.3131	0.3156	0.0382	0.027*	
O13W	0.6077 (5)	0.4766 (5)	0.0792 (2)	0.0170 (12)	
H13A	0.5905	0.5421	0.0898	0.025*	
H13B	0.5972	0.4646	0.0437	0.025*	
O14W	0.7541 (5)	0.5871 (5)	0.1854 (2)	0.0174 (12)	
H14A	0.7569	0.6633	0.1802	0.026*	
H14B	0.6802	0.5398	0.1954	0.026*	
O15W	1.0571 (5)	0.7556 (5)	0.2066 (2)	0.0243 (14)	
H15A	1.0246	0.7608	0.1733	0.036*	
H15B	1.1339	0.7524	0.2075	0.036*	
O16W	0.9349 (5)	0.7502 (5)	0.2956 (2)	0.0228 (14)	
H16A	0.8580	0.7534	0.2946	0.027*	
H16B	0.9671	0.7449	0.3289	0.027*	
O17W	0.9596 (5)	0.4918 (6)	0.1720 (2)	0.0279 (15)	
H17A	1.0350	0.5346	0.1622	0.042*	
H17B	0.9514	0.4142	0.1778	0.042*	
O18W	0.0315 (5)	1.0158 (5)	0.3297 (2)	0.0209 (13)	
H18A	0.0396	1.0934	0.3238	0.031*	
H18B	−0.0434	0.9730	0.3399	0.031*	
O19W	0.2389 (5)	0.9159 (5)	0.3135 (2)	0.0183 (13)	
H19A	0.3121	0.9625	0.3029	0.027*	
H19B	0.2354	0.8393	0.3185	0.027*	
O20W	0.3058 (5)	1.1735 (5)	0.3436 (2)	0.0173 (12)	
H20A	0.2959	1.1550	0.3083	0.026*	
H20B	0.2893	1.2409	0.3515	0.026*	
O21W	0.5954 (5)	1.3463 (5)	0.3459 (2)	0.0210 (13)	
H21A	0.5822	1.3258	0.3106	0.032*	
H21B	0.5828	1.4160	0.3539	0.032*	
O22W	0.3846 (5)	1.0217 (5)	0.4204 (2)	0.0161 (12)	
H22A	0.3965	1.0342	0.4560	0.024*	
H22B	0.3996	0.9551	0.4094	0.024*	
O23W	0.6749 (5)	1.1915 (5)	0.4225 (2)	0.0183 (13)	
H23A	0.6906	1.2049	0.4582	0.027*	
H23B	0.6860	1.1230	0.4109	0.027*	
O24W	0.7492 (8)	1.4650 (6)	0.4535 (3)	0.050 (2)	
H24A	0.7406	1.5324	0.4417	0.075*	
H24B	0.6753	1.4174	0.4633	0.075*	
O25W	0.9619 (6)	1.3470 (7)	0.4175 (3)	0.0397 (18)	
H25A	1.0010	1.3506	0.3895	0.059*	
H25B	0.9422	1.2695	0.4266	0.059*	
O26W	0.0268 (7)	0.6045 (7)	0.4655 (3)	0.055 (2)	
H26A	−0.0103	0.5943	0.4312	0.082*	
H26B	0.0808	0.6852	0.4767	0.082*	
O27W	0.0246 (7)	0.1633 (7)	0.0101 (3)	0.047 (2)	
H27A	0.0433	0.2452	0.0132	0.070*	
H27B	−0.0572	0.1211	0.0126	0.070*	
O28W	−0.0237 (6)	−0.0493 (7)	0.0732 (3)	0.0438 (19)	
H28A	−0.0259	−0.1086	0.0490	0.066*	
H28B	−0.0949	−0.0364	0.0655	0.066*	
O29W	0.2076 (5)	0.0083 (5)	0.0286 (2)	0.0237 (14)	
H29A	0.2398	−0.0317	0.0508	0.036*	
H29B	0.2678	0.0544	0.0115	0.036*	
O30W	0.4040 (5)	0.1487 (5)	0.1495 (2)	0.0260 (15)	
H30A	0.3196	0.1262	0.1414	0.039*	
H30B	0.4302	0.1690	0.1845	0.039*	
O31W	0.5088 (5)	0.4329 (5)	0.1786 (2)	0.0239 (14)	
H31A	0.4989	0.3920	0.2067	0.036*	
H31B	0.4461	0.4589	0.1715	0.036*	
O32W	0.1998 (6)	1.0926 (6)	0.2446 (2)	0.0375 (18)	
H32A	0.1630	1.1466	0.2458	0.056*	
H32B	0.2152	1.0820	0.2121	0.056*	
O33W	0.0827 (5)	0.8155 (5)	0.3844 (2)	0.0197 (13)	
H33A	0.0498	0.8084	0.4139	0.030*	
H33B	0.1310	0.7721	0.3844	0.030*	
O34W	0.7955 (7)	1.2617 (6)	0.3126 (3)	0.0394 (18)	
H34A	0.7285	1.1878	0.3033	0.059*	
H34B	0.8677	1.2499	0.3175	0.059*	
O35W	0.9524 (6)	1.1264 (6)	0.4358 (3)	0.0367 (17)	
O36W	0.0339 (7)	0.3635 (6)	0.0656 (3)	0.044 (2)	
O37W	0.4813 (7)	1.2430 (8)	0.2503 (3)	0.047 (2)	
O38W	0.1417 (7)	0.8435 (8)	0.4863 (3)	0.057 (2)	
O39W	0.0156 (8)	0.2317 (7)	0.3108 (3)	0.059 (2)	
O40W	0.1094 (9)	0.2640 (8)	0.2269 (3)	0.073 (3)	

### Atomic displacement parameters (Å^2^)

**Table:**

	*U*^11^	*U*^22^	*U*^33^	*U*^12^	*U*^13^	*U*^23^
Pt1	0.00654 (13)	0.00628 (15)	0.00492 (15)	0.00380 (11)	0.00147 (10)	0.00062 (11)
V1	0.0093 (6)	0.0106 (7)	0.0091 (7)	0.0024 (5)	0.0014 (5)	0.0012 (5)
V2	0.0090 (6)	0.0123 (7)	0.0117 (7)	0.0048 (5)	0.0006 (5)	0.0015 (5)
V3	0.0111 (6)	0.0116 (7)	0.0145 (7)	0.0044 (5)	0.0044 (5)	0.0046 (6)
V4	0.0099 (6)	0.0100 (6)	0.0075 (6)	0.0062 (5)	0.0014 (5)	0.0020 (5)
V5	0.0102 (6)	0.0138 (7)	0.0108 (7)	0.0063 (5)	0.0004 (5)	0.0010 (6)
V6	0.0111 (6)	0.0097 (6)	0.0072 (6)	0.0049 (5)	0.0017 (5)	−0.0013 (5)
V7	0.0105 (6)	0.0112 (7)	0.0088 (7)	0.0055 (5)	0.0042 (5)	0.0001 (5)
V8	0.0115 (6)	0.0079 (6)	0.0125 (7)	0.0034 (5)	0.0038 (5)	−0.0004 (5)
V9	0.0098 (6)	0.0098 (6)	0.0089 (7)	0.0053 (5)	0.0021 (5)	−0.0013 (5)
O1D	0.008 (2)	0.008 (3)	0.004 (3)	0.003 (2)	0.0004 (18)	−0.001 (2)
O2D	0.010 (2)	0.011 (3)	0.011 (3)	0.005 (2)	0.004 (2)	0.003 (2)
O3C	0.009 (2)	0.005 (3)	0.008 (3)	0.003 (2)	0.0015 (19)	−0.004 (2)
O4C	0.010 (2)	0.009 (3)	0.009 (3)	0.005 (2)	0.003 (2)	0.002 (2)
O5C	0.011 (2)	0.010 (3)	0.006 (3)	0.007 (2)	0.0017 (19)	0.002 (2)
O6C	0.009 (2)	0.009 (3)	0.008 (3)	0.003 (2)	0.0025 (19)	0.005 (2)
O8B	0.008 (2)	0.016 (3)	0.008 (3)	0.007 (2)	0.003 (2)	0.002 (2)
O9B	0.011 (2)	0.010 (3)	0.015 (3)	0.004 (2)	0.003 (2)	0.000 (2)
O10B	0.013 (2)	0.009 (3)	0.012 (3)	0.006 (2)	0.003 (2)	0.004 (2)
O11B	0.009 (2)	0.014 (3)	0.012 (3)	0.008 (2)	0.000 (2)	0.001 (2)
O12B	0.010 (2)	0.012 (3)	0.009 (3)	0.002 (2)	0.001 (2)	−0.002 (2)
O13B	0.012 (2)	0.010 (3)	0.008 (3)	0.001 (2)	0.000 (2)	−0.001 (2)
O14B	0.010 (2)	0.017 (3)	0.015 (3)	0.010 (2)	0.003 (2)	0.003 (2)
O15B	0.011 (2)	0.016 (3)	0.015 (3)	0.007 (2)	0.003 (2)	0.003 (2)
O16B	0.014 (3)	0.012 (3)	0.011 (3)	0.005 (2)	0.008 (2)	0.002 (2)
O17B	0.014 (3)	0.008 (3)	0.007 (3)	0.005 (2)	−0.001 (2)	−0.002 (2)
O18B	0.012 (3)	0.014 (3)	0.018 (3)	0.006 (2)	0.008 (2)	0.008 (2)
O19B	0.013 (3)	0.011 (3)	0.015 (3)	0.006 (2)	0.003 (2)	0.005 (2)
O20B	0.009 (2)	0.013 (3)	0.015 (3)	0.007 (2)	0.002 (2)	0.001 (2)
O21T	0.012 (3)	0.009 (3)	0.017 (3)	0.001 (2)	0.004 (2)	0.003 (2)
O22T	0.015 (3)	0.019 (3)	0.016 (3)	0.010 (2)	0.002 (2)	0.003 (2)
O23T	0.018 (3)	0.014 (3)	0.023 (3)	0.004 (2)	0.007 (2)	0.008 (3)
O24T	0.016 (3)	0.028 (4)	0.016 (3)	0.011 (3)	0.000 (2)	0.003 (3)
O25T	0.019 (3)	0.014 (3)	0.010 (3)	0.006 (2)	0.004 (2)	−0.004 (2)
O26T	0.015 (3)	0.019 (3)	0.017 (3)	0.011 (2)	0.006 (2)	−0.002 (2)
O27T	0.018 (3)	0.017 (3)	0.019 (3)	0.009 (2)	0.005 (2)	−0.002 (2)
O28T	0.016 (3)	0.015 (3)	0.018 (3)	0.009 (2)	0.007 (2)	−0.002 (2)
Pt2	0.00667 (13)	0.00671 (15)	0.00512 (15)	0.00362 (11)	0.00141 (10)	0.00057 (11)
V10	0.0097 (6)	0.0157 (7)	0.0116 (7)	0.0056 (5)	0.0016 (5)	0.0026 (6)
V11	0.0106 (6)	0.0118 (7)	0.0104 (7)	0.0026 (5)	0.0024 (5)	0.0020 (5)
V12	0.0096 (6)	0.0140 (7)	0.0109 (7)	0.0058 (5)	0.0006 (5)	0.0008 (6)
V13	0.0105 (6)	0.0097 (6)	0.0079 (6)	0.0063 (5)	0.0022 (5)	0.0022 (5)
V14	0.0127 (6)	0.0102 (7)	0.0148 (7)	0.0057 (5)	0.0052 (5)	0.0036 (6)
V15	0.0124 (6)	0.0105 (7)	0.0088 (7)	0.0067 (5)	0.0044 (5)	0.0010 (5)
V16	0.0111 (6)	0.0102 (7)	0.0080 (6)	0.0048 (5)	0.0012 (5)	−0.0009 (5)
V17	0.0101 (6)	0.0124 (7)	0.0092 (7)	0.0051 (5)	0.0041 (5)	0.0001 (5)
V18	0.0125 (6)	0.0076 (6)	0.0102 (7)	0.0039 (5)	0.0027 (5)	−0.0010 (5)
O29D	0.008 (2)	0.009 (3)	0.009 (3)	0.003 (2)	0.003 (2)	0.001 (2)
O30D	0.007 (2)	0.008 (3)	0.012 (3)	0.004 (2)	0.0003 (19)	0.000 (2)
O31C	0.011 (2)	0.007 (3)	0.011 (3)	0.006 (2)	0.004 (2)	0.003 (2)
O32C	0.013 (2)	0.011 (3)	0.005 (3)	0.006 (2)	0.000 (2)	−0.002 (2)
O33C	0.011 (2)	0.013 (3)	0.010 (3)	0.006 (2)	0.003 (2)	0.003 (2)
O34C	0.010 (2)	0.009 (3)	0.008 (3)	0.004 (2)	−0.001 (2)	0.001 (2)
O35B	0.009 (2)	0.012 (3)	0.009 (3)	0.004 (2)	0.001 (2)	0.001 (2)
O36B	0.015 (3)	0.009 (3)	0.006 (2)	0.004 (2)	0.004 (2)	0.003 (2)
O37B	0.009 (2)	0.013 (3)	0.010 (3)	0.001 (2)	−0.002 (2)	−0.001 (2)
O38B	0.011 (2)	0.014 (3)	0.009 (3)	0.005 (2)	0.002 (2)	0.002 (2)
O39B	0.013 (3)	0.016 (3)	0.009 (3)	0.008 (2)	0.002 (2)	0.003 (2)
O40B	0.010 (2)	0.015 (3)	0.013 (3)	0.003 (2)	0.004 (2)	0.000 (2)
O41B	0.011 (2)	0.013 (3)	0.010 (3)	0.006 (2)	0.004 (2)	0.002 (2)
O42B	0.016 (3)	0.011 (3)	0.004 (3)	0.005 (2)	−0.002 (2)	0.001 (2)
O43B	0.012 (2)	0.014 (3)	0.011 (3)	0.003 (2)	0.001 (2)	0.002 (2)
O44B	0.015 (3)	0.012 (3)	0.008 (3)	0.006 (2)	−0.004 (2)	−0.001 (2)
O45B	0.011 (2)	0.012 (3)	0.012 (3)	0.005 (2)	0.005 (2)	0.001 (2)
O46B	0.010 (2)	0.017 (3)	0.012 (3)	0.007 (2)	0.000 (2)	0.003 (2)
O47B	0.013 (3)	0.020 (3)	0.011 (3)	0.011 (2)	0.002 (2)	0.003 (2)
O48B	0.015 (3)	0.009 (3)	0.013 (3)	0.005 (2)	0.005 (2)	0.001 (2)
O49T	0.014 (3)	0.022 (3)	0.022 (3)	0.006 (2)	0.002 (2)	0.008 (3)
O50T	0.021 (3)	0.020 (3)	0.012 (3)	0.007 (2)	0.003 (2)	0.007 (2)
O51T	0.012 (3)	0.024 (3)	0.017 (3)	0.010 (2)	−0.001 (2)	0.001 (3)
O52T	0.021 (3)	0.015 (3)	0.020 (3)	0.008 (2)	0.008 (2)	0.007 (3)
O53T	0.024 (3)	0.016 (3)	0.011 (3)	0.012 (2)	0.008 (2)	0.005 (2)
O54T	0.020 (3)	0.013 (3)	0.011 (3)	0.008 (2)	0.003 (2)	−0.001 (2)
O55T	0.023 (3)	0.016 (3)	0.014 (3)	0.007 (2)	0.004 (2)	0.000 (2)
O56T	0.020 (3)	0.019 (3)	0.013 (3)	0.011 (2)	0.003 (2)	−0.003 (2)
Na1	0.035 (2)	0.027 (2)	0.040 (2)	0.0101 (17)	0.0161 (18)	0.0027 (18)
Na2	0.0203 (16)	0.0149 (17)	0.0182 (18)	0.0082 (14)	0.0041 (13)	0.0035 (14)
Na3	0.0195 (16)	0.0193 (18)	0.0210 (19)	0.0094 (14)	0.0050 (14)	0.0011 (15)
Na4	0.0174 (15)	0.0157 (17)	0.0167 (18)	0.0082 (13)	0.0003 (13)	−0.0009 (14)
Na5	0.0170 (15)	0.0152 (17)	0.0138 (17)	0.0063 (13)	0.0024 (12)	0.0009 (13)
Na6	0.0232 (18)	0.0217 (18)	0.039 (2)	0.0115 (15)	0.0097 (16)	0.0091 (16)
Na7	0.0176 (16)	0.0214 (18)	0.0189 (18)	0.0072 (14)	0.0011 (13)	0.0009 (14)
Na8	0.0191 (16)	0.0192 (18)	0.0192 (18)	0.0078 (14)	0.0001 (13)	0.0018 (14)
Na9	0.047 (3)	0.060 (3)	0.051 (3)	0.023 (2)	0.017 (2)	0.009 (2)
O1W	0.031 (4)	0.031 (4)	0.050 (5)	0.010 (3)	0.009 (3)	−0.001 (3)
O2W	0.060 (5)	0.021 (4)	0.047 (5)	0.028 (4)	0.024 (4)	0.007 (3)
O3W	0.111 (7)	0.028 (4)	0.006 (3)	0.044 (5)	0.007 (4)	0.004 (3)
O4W	0.023 (3)	0.032 (4)	0.025 (4)	0.019 (3)	0.012 (3)	0.011 (3)
O5W	0.047 (4)	0.024 (4)	0.011 (3)	0.013 (3)	−0.002 (3)	−0.003 (3)
O6W	0.025 (3)	0.015 (3)	0.022 (3)	0.011 (3)	0.001 (3)	−0.002 (3)
O7W	0.028 (3)	0.027 (4)	0.024 (4)	0.014 (3)	0.010 (3)	0.003 (3)
O8W	0.046 (4)	0.029 (4)	0.022 (4)	0.021 (3)	0.005 (3)	0.007 (3)
O9W	0.049 (4)	0.026 (4)	0.015 (3)	0.026 (3)	0.007 (3)	0.000 (3)
O10W	0.093 (7)	0.046 (5)	0.026 (4)	0.052 (5)	0.017 (4)	0.015 (4)
O11W	0.033 (4)	0.020 (3)	0.024 (3)	0.011 (3)	0.010 (3)	0.005 (3)
O12W	0.024 (3)	0.013 (3)	0.018 (3)	0.008 (2)	0.005 (2)	0.001 (2)
O13W	0.021 (3)	0.018 (3)	0.016 (3)	0.012 (2)	0.005 (2)	0.003 (2)
O14W	0.018 (3)	0.019 (3)	0.018 (3)	0.011 (2)	0.005 (2)	0.004 (3)
O15W	0.018 (3)	0.027 (4)	0.026 (4)	0.006 (3)	0.009 (3)	0.000 (3)
O16W	0.017 (3)	0.026 (3)	0.025 (4)	0.007 (3)	0.010 (3)	0.003 (3)
O17W	0.021 (3)	0.028 (4)	0.033 (4)	0.010 (3)	0.003 (3)	−0.001 (3)
O18W	0.017 (3)	0.023 (3)	0.026 (3)	0.012 (3)	0.003 (2)	0.003 (3)
O19W	0.018 (3)	0.017 (3)	0.022 (3)	0.008 (2)	0.007 (2)	0.004 (3)
O20W	0.020 (3)	0.013 (3)	0.022 (3)	0.011 (2)	0.003 (2)	0.001 (2)
O21W	0.024 (3)	0.018 (3)	0.025 (3)	0.012 (3)	0.006 (3)	0.007 (3)
O22W	0.019 (3)	0.019 (3)	0.014 (3)	0.013 (2)	0.001 (2)	0.004 (2)
O23W	0.018 (3)	0.016 (3)	0.022 (3)	0.008 (2)	0.002 (2)	0.001 (3)
O24W	0.089 (6)	0.039 (4)	0.060 (5)	0.046 (5)	0.061 (5)	0.037 (4)
O25W	0.031 (4)	0.051 (5)	0.051 (5)	0.025 (4)	0.016 (3)	0.031 (4)
O26W	0.044 (5)	0.048 (5)	0.069 (6)	0.018 (4)	0.000 (4)	0.019 (5)
O27W	0.050 (5)	0.035 (4)	0.053 (5)	0.019 (4)	0.002 (4)	0.002 (4)
O28W	0.031 (4)	0.049 (5)	0.057 (5)	0.020 (4)	0.016 (4)	0.012 (4)
O29W	0.030 (3)	0.032 (4)	0.020 (3)	0.019 (3)	0.014 (3)	0.014 (3)
O30W	0.026 (3)	0.022 (3)	0.033 (4)	0.013 (3)	0.008 (3)	0.005 (3)
O31W	0.033 (3)	0.023 (3)	0.021 (4)	0.018 (3)	0.004 (3)	−0.003 (3)
O32W	0.043 (4)	0.035 (4)	0.023 (4)	0.005 (3)	0.004 (3)	−0.001 (3)
O33W	0.023 (3)	0.019 (3)	0.022 (3)	0.011 (3)	0.010 (2)	0.005 (3)
O34W	0.052 (5)	0.025 (4)	0.041 (4)	0.018 (3)	−0.001 (4)	−0.001 (3)
O35W	0.033 (4)	0.029 (4)	0.038 (4)	0.007 (3)	−0.005 (3)	−0.001 (3)
O36W	0.052 (5)	0.026 (4)	0.044 (5)	0.016 (4)	−0.014 (4)	−0.009 (4)
O37W	0.061 (5)	0.069 (5)	0.031 (4)	0.054 (5)	0.002 (4)	−0.009 (4)
O38W	0.044 (5)	0.065 (6)	0.059 (6)	0.023 (4)	0.003 (4)	−0.003 (5)
O39W	0.058 (6)	0.034 (5)	0.081 (7)	0.013 (4)	0.013 (5)	0.013 (5)
O40W	0.075 (7)	0.045 (5)	0.064 (7)	−0.002 (5)	−0.015 (5)	0.015 (5)

### Geometric parameters (Å, º)

**Table:**

Pt1—O1D	1.970 (5)	V17—O34C	2.035 (5)
Pt1—O2D	1.982 (5)	V17—O30D	2.265 (5)
Pt1—O4C	2.012 (5)	V17—V18	3.1050 (18)
Pt1—O7B	2.012 (5)	V18—O55T	1.603 (6)
Pt1—O3C	2.025 (5)	V18—O48B	1.779 (5)
Pt1—O8B	2.025 (5)	V18—O44B	1.907 (5)
Pt1—V8	3.1038 (14)	V18—O34C	1.942 (5)
Pt1—V6	3.1065 (13)	V18—O32C	2.057 (5)
Pt1—V7	3.1239 (13)	V18—O29D	2.253 (5)
Pt1—V9	3.1393 (13)	O35B—H35	0.85 (3)
Pt1—V1	3.1495 (13)	O36B—H36	0.84 (3)
Pt1—V4	3.1627 (13)	O44B—H44	0.84 (3)
V1—O21T	1.603 (5)	O49T—Na8^ii^	2.420 (7)
V1—O12B	1.830 (5)	Na1—O29W	2.353 (7)
V1—O16B	1.882 (5)	Na1—O27W	2.360 (9)
V1—O13B	1.882 (5)	Na1—O12W	2.379 (7)
V1—O7B	2.058 (5)	Na1—O1W	2.421 (8)
V1—O1D	2.340 (5)	Na1—O28W	2.448 (8)
V1—V5	3.1109 (19)	Na1—O2W	2.742 (9)
V2—O22T	1.604 (5)	Na2—O3W	2.361 (7)
V2—O9B	1.832 (6)	Na2—O30W	2.364 (7)
V2—O14B	1.841 (6)	Na2—O31W	2.372 (7)
V2—O15B	1.904 (5)	Na2—O13W	2.378 (6)
V2—O8B	2.076 (5)	Na2—O6W	2.425 (7)
V2—O2D	2.387 (5)	Na2—O12W	2.488 (7)
V2—V3	3.1190 (19)	Na2—Na3	3.506 (4)
V3—O23T	1.595 (6)	Na3—O5W	2.355 (7)
V3—O9B	1.837 (5)	Na3—O13W	2.387 (6)
V3—O19B	1.840 (5)	Na3—O4W	2.399 (7)
V3—O18B	1.883 (6)	Na3—O14W	2.407 (6)
V3—O10B	2.054 (5)	Na3—O17W	2.409 (7)
V3—O2D	2.382 (5)	Na3—O6W	2.429 (7)
V3—V4	3.1027 (18)	Na4—O8W	2.358 (8)
V4—O10B	1.674 (5)	Na4—O22W	2.387 (6)
V4—O11B	1.675 (5)	Na4—O20W	2.397 (7)
V4—O6C	1.916 (5)	Na4—O33W	2.400 (7)
V4—O5C	1.922 (5)	Na4—O18W	2.436 (6)
V4—O2D	2.159 (5)	Na4—O19W	2.441 (6)
V4—O1D	2.174 (5)	Na5—O9W	2.339 (7)
V5—O24T	1.578 (6)	Na5—O10W	2.357 (8)
V5—O17B	1.838 (5)	Na5—O22W	2.382 (6)
V5—O12B	1.842 (6)	Na5—O20W	2.389 (6)
V5—O20B	1.896 (5)	Na5—O23W	2.418 (6)
V5—O11B	2.059 (5)	Na5—O21W	2.425 (7)
V5—O1D	2.419 (5)	Na5—H10B	2.59 (12)
V6—O25T	1.602 (5)	Na6—O23W	2.375 (7)
V6—O13B	1.805 (5)	Na6—O11W	2.380 (7)
V6—O17B	1.843 (5)	Na6—O25W	2.390 (7)
V6—O3C	2.001 (5)	Na6—O21W	2.417 (7)
V6—O5C	2.023 (5)	Na6—O24W	2.436 (8)
V6—O1D	2.271 (5)	Na6—O34W	2.444 (8)
V6—V7	3.1090 (18)	Na7—O7W	2.385 (7)
V7—O26T	1.610 (5)	Na7—O14W	2.399 (6)
V7—O18B	1.802 (5)	Na7—O15W	2.419 (7)
V7—O14B	1.847 (5)	Na7—O16W	2.432 (7)
V7—O5C	2.007 (5)	Na7—O17W	2.432 (7)
V7—O3C	2.036 (5)	Na7—O22T^ii^	2.454 (6)
V7—O2D	2.255 (5)	Na8—O19W	2.373 (6)
V8—O27T	1.599 (6)	Na8—O49T^i^	2.420 (7)
V8—O15B	1.808 (5)	Na8—O16W^i^	2.428 (7)
V8—O19B	1.840 (5)	Na8—O32W	2.428 (8)
V8—O4C	2.024 (5)	Na8—O15W^i^	2.431 (7)
V8—O6C	2.026 (5)	Na8—O18W	2.444 (7)
V8—O2D	2.268 (5)	Na9—O24W^iii^	2.204 (11)
V9—O28T	1.603 (5)	Na9—O26W	2.319 (9)
V9—O20B	1.805 (5)	Na9—O26W^iv^	2.395 (10)
V9—O16B	1.851 (5)	Na9—O25W^v^	2.410 (9)
V9—O6C	1.987 (5)	O1W—H1A	0.92 (3)
V9—O4C	2.056 (5)	O1W—H1B	0.90 (3)
V9—O1D	2.260 (5)	O2W—H2A	0.90 (3)
O7B—H7	0.85 (3)	O2W—H2B	0.88 (3)
O8B—H8	0.86 (3)	O3W—H3A	0.86 (3)
O15B—O50T	2.889 (8)	O3W—H3B	0.87 (3)
O18B—Na9	2.498 (7)	O4W—H4A	0.84 (3)
O22T—Na7^i^	2.454 (6)	O4W—H4B	0.84 (3)
O23T—Na9	2.847 (7)	O5W—H5A	0.87 (3)
Pt2—O29D	1.966 (5)	O5W—H5B	0.86 (3)
Pt2—O30D	1.980 (5)	O6W—H6A	0.85 (3)
Pt2—O32C	2.010 (5)	O6W—H6B	0.84 (3)
Pt2—O35B	2.015 (5)	O7W—H7A	0.83 (3)
Pt2—O31C	2.017 (5)	O7W—H7B	0.84 (3)
Pt2—O36B	2.017 (5)	O8W—H8A	0.86 (3)
Pt2—V17	3.1101 (13)	O8W—H8B	0.85 (3)
Pt2—V15	3.1193 (13)	O9W—H9A	0.85 (3)
Pt2—V16	3.1202 (13)	O9W—H9B	0.84 (3)
Pt2—V18	3.1413 (14)	O10W—H10A	0.88 (3)
Pt2—V10	3.1485 (13)	O10W—H10B	0.87 (3)
Pt2—V11	3.1613 (13)	O11W—H11A	0.87 (3)
V10—O49T	1.590 (6)	O11W—H11B	0.89 (3)
V10—O41B	1.820 (5)	O12W—H12A	0.9900
V10—O40B	1.841 (6)	O12W—H12B	0.9900
V10—O44B	1.950 (6)	O13W—H13A	0.9900
V10—O35B	2.059 (5)	O13W—H13B	0.9900
V10—O29D	2.340 (5)	O14W—H14A	0.9900
V10—V14	3.1158 (19)	O14W—H14B	0.9900
V11—O50T	1.585 (6)	O15W—H15A	0.9900
V11—O37B	1.846 (5)	O15W—H15B	0.9900
V11—O42B	1.882 (5)	O16W—H16A	0.9900
V11—O43B	1.905 (6)	O16W—H16B	0.9900
V11—O36B	2.035 (5)	O17W—H17A	0.9900
V11—O30D	2.399 (5)	O17W—H17B	0.9900
V12—O51T	1.596 (5)	O18W—H18A	0.9900
V12—O37B	1.813 (6)	O18W—H18B	0.9900
V12—O46B	1.853 (5)	O19W—H19A	0.9900
V12—O47B	1.869 (6)	O19W—H19B	0.9900
V12—O38B	2.103 (5)	O20W—H20A	0.9900
V12—O30D	2.408 (5)	O20W—H20B	0.9900
V13—O38B	1.666 (5)	O21W—H21A	0.9900
V13—O39B	1.683 (5)	O21W—H21B	0.9900
V13—O33C	1.919 (5)	O22W—H22A	0.9900
V13—O34C	1.929 (5)	O22W—H22B	0.9900
V13—O30D	2.136 (5)	O23W—H23A	0.9900
V13—O29D	2.215 (5)	O23W—H23B	0.9900
V14—O52T	1.583 (6)	O24W—H24A	0.9900
V14—O40B	1.819 (5)	O24W—H24B	0.9900
V14—O45B	1.854 (5)	O25W—H25A	0.9900
V14—O48B	1.904 (5)	O25W—H25B	0.9900
V14—O39B	2.048 (5)	O26W—H26A	0.9900
V14—O29D	2.383 (5)	O26W—H26B	0.9900
V15—O53T	1.591 (5)	O27W—H27A	0.9800
V15—O45B	1.824 (5)	O27W—H27B	0.9800
V15—O41B	1.859 (5)	O28W—H28A	0.9800
V15—O33C	1.989 (5)	O28W—H28B	0.9800
V15—O31C	1.992 (5)	O29W—H29A	0.9800
V15—O29D	2.302 (5)	O29W—H29B	0.9800
V15—V16	3.0899 (18)	O30W—H30A	0.9800
V16—O54T	1.603 (5)	O30W—H30B	0.9800
V16—O42B	1.808 (5)	O31W—H31A	0.9800
V16—O46B	1.834 (5)	O31W—H31B	0.9800
V16—O31C	2.032 (5)	O32W—H32A	0.9800
V16—O33C	2.033 (5)	O32W—H32B	0.9800
V16—O30D	2.260 (5)	O33W—H33A	0.9800
V17—O56T	1.601 (5)	O33W—H33B	0.9800
V17—O47B	1.821 (5)	O34W—H34A	0.9801
V17—O43B	1.822 (6)	O34W—H34B	0.9801
V17—O32C	2.027 (5)		
			
O1D—Pt1—O2D	85.0 (2)	O40B—V10—O35B	157.9 (2)
O1D—Pt1—O4C	85.4 (2)	O44B—V10—O35B	83.5 (2)
O2D—Pt1—O4C	86.0 (2)	O49T—V10—O29D	174.4 (3)
O1D—Pt1—O7B	87.7 (2)	O41B—V10—O29D	77.4 (2)
O2D—Pt1—O7B	172.5 (2)	O40B—V10—O29D	80.7 (2)
O4C—Pt1—O7B	95.1 (2)	O44B—V10—O29D	73.9 (2)
O1D—Pt1—O3C	85.4 (2)	O35B—V10—O29D	77.41 (19)
O2D—Pt1—O3C	85.2 (2)	O49T—V10—V14	135.4 (2)
O4C—Pt1—O3C	167.8 (2)	O41B—V10—V14	83.73 (17)
O7B—Pt1—O3C	92.5 (2)	O40B—V10—V14	31.43 (16)
O1D—Pt1—O8B	173.5 (2)	O44B—V10—V14	81.89 (17)
O2D—Pt1—O8B	88.5 (2)	O35B—V10—V14	126.73 (15)
O4C—Pt1—O8B	94.3 (2)	O29D—V10—V14	49.32 (13)
O7B—Pt1—O8B	98.8 (2)	O49T—V10—Pt2	136.6 (2)
O3C—Pt1—O8B	93.9 (2)	O41B—V10—Pt2	77.74 (16)
O1D—Pt1—V8	89.71 (15)	O40B—V10—Pt2	119.20 (17)
O2D—Pt1—V8	46.83 (15)	O44B—V10—Pt2	76.50 (16)
O4C—Pt1—V8	39.88 (15)	O35B—V10—Pt2	38.88 (14)
O7B—Pt1—V8	134.92 (15)	O29D—V10—Pt2	38.55 (13)
O3C—Pt1—V8	132.08 (15)	V14—V10—Pt2	87.86 (4)
O8B—Pt1—V8	85.95 (16)	O50T—V11—O37B	102.3 (3)
O1D—Pt1—V6	46.84 (14)	O50T—V11—O42B	103.7 (3)
O2D—Pt1—V6	89.20 (15)	O37B—V11—O42B	91.4 (2)
O4C—Pt1—V6	132.19 (14)	O50T—V11—O43B	104.1 (3)
O7B—Pt1—V6	84.60 (15)	O37B—V11—O43B	90.0 (2)
O3C—Pt1—V6	39.23 (14)	O42B—V11—O43B	151.2 (2)
O8B—Pt1—V6	133.10 (15)	O50T—V11—O36B	99.6 (3)
V8—Pt1—V6	123.87 (4)	O37B—V11—O36B	158.1 (2)
O1D—Pt1—V7	89.07 (14)	O42B—V11—O36B	83.3 (2)
O2D—Pt1—V7	46.00 (15)	O43B—V11—O36B	84.7 (2)
O4C—Pt1—V7	131.97 (15)	O50T—V11—O30D	176.9 (3)
O7B—Pt1—V7	132.36 (15)	O37B—V11—O30D	80.8 (2)
O3C—Pt1—V7	39.83 (15)	O42B—V11—O30D	75.7 (2)
O8B—Pt1—V7	86.34 (15)	O43B—V11—O30D	76.2 (2)
V8—Pt1—V7	92.55 (3)	O36B—V11—O30D	77.27 (19)
V6—Pt1—V7	59.87 (3)	O50T—V11—Pt2	138.2 (2)
O1D—Pt1—V9	45.75 (14)	O37B—V11—Pt2	119.53 (17)
O2D—Pt1—V9	88.58 (14)	O42B—V11—Pt2	77.15 (16)
O4C—Pt1—V9	40.01 (14)	O43B—V11—Pt2	77.21 (16)
O7B—Pt1—V9	87.51 (15)	O36B—V11—Pt2	38.53 (15)
O3C—Pt1—V9	131.16 (14)	O30D—V11—Pt2	38.74 (12)
O8B—Pt1—V9	134.34 (15)	O51T—V12—O37B	105.4 (3)
V8—Pt1—V9	60.05 (3)	O51T—V12—O46B	104.2 (3)
V6—Pt1—V9	92.40 (3)	O37B—V12—O46B	91.9 (2)
V7—Pt1—V9	121.82 (3)	O51T—V12—O47B	104.1 (3)
O1D—Pt1—V1	47.87 (15)	O37B—V12—O47B	91.5 (2)
O2D—Pt1—V1	132.90 (15)	O46B—V12—O47B	149.5 (2)
O4C—Pt1—V1	89.57 (14)	O51T—V12—O38B	100.7 (3)
O7B—Pt1—V1	39.84 (15)	O37B—V12—O38B	153.9 (2)
O3C—Pt1—V1	90.19 (14)	O46B—V12—O38B	82.4 (2)
O8B—Pt1—V1	138.59 (15)	O47B—V12—O38B	81.4 (2)
V8—Pt1—V1	120.36 (3)	O51T—V12—O30D	173.4 (3)
V6—Pt1—V1	60.49 (3)	O37B—V12—O30D	81.2 (2)
V7—Pt1—V1	120.36 (3)	O46B—V12—O30D	75.2 (2)
V9—Pt1—V1	60.34 (3)	O47B—V12—O30D	75.5 (2)
O1D—Pt1—V4	42.71 (15)	O38B—V12—O30D	72.73 (19)
O2D—Pt1—V4	42.31 (14)	O38B—V13—O39B	108.8 (3)
O4C—Pt1—V4	84.03 (15)	O38B—V13—O33C	99.2 (2)
O7B—Pt1—V4	130.38 (15)	O39B—V13—O33C	97.2 (2)
O3C—Pt1—V4	83.75 (15)	O38B—V13—O34C	98.1 (2)
O8B—Pt1—V4	130.82 (15)	O39B—V13—O34C	97.4 (2)
V8—Pt1—V4	61.95 (3)	O33C—V13—O34C	152.3 (2)
V6—Pt1—V4	61.92 (3)	O38B—V13—O30D	89.0 (2)
V7—Pt1—V4	61.13 (3)	O39B—V13—O30D	162.2 (2)
V9—Pt1—V4	60.69 (3)	O33C—V13—O30D	79.4 (2)
V1—Pt1—V4	90.58 (3)	O34C—V13—O30D	79.5 (2)
O21T—V1—O12B	102.9 (3)	O38B—V13—O29D	164.9 (2)
O21T—V1—O16B	102.5 (3)	O39B—V13—O29D	86.3 (2)
O12B—V1—O16B	91.3 (2)	O33C—V13—O29D	79.6 (2)
O21T—V1—O13B	104.3 (3)	O34C—V13—O29D	78.1 (2)
O12B—V1—O13B	91.2 (2)	O30D—V13—O29D	75.97 (19)
O16B—V1—O13B	151.8 (2)	O38B—V13—Pt2	126.97 (19)
O21T—V1—O7B	97.3 (2)	O39B—V13—Pt2	124.23 (18)
O12B—V1—O7B	159.8 (2)	O33C—V13—Pt2	76.42 (16)
O16B—V1—O7B	85.8 (2)	O34C—V13—Pt2	75.92 (16)
O13B—V1—O7B	82.2 (2)	O30D—V13—Pt2	38.00 (13)
O21T—V1—O1D	174.6 (2)	O29D—V13—Pt2	37.97 (13)
O12B—V1—O1D	82.5 (2)	O52T—V14—O40B	103.9 (3)
O16B—V1—O1D	76.5 (2)	O52T—V14—O45B	103.4 (3)
O13B—V1—O1D	75.9 (2)	O40B—V14—O45B	91.3 (2)
O7B—V1—O1D	77.38 (19)	O52T—V14—O48B	103.8 (3)
O21T—V1—V5	135.1 (2)	O40B—V14—O48B	90.9 (2)
O12B—V1—V5	32.21 (17)	O45B—V14—O48B	151.2 (2)
O16B—V1—V5	84.60 (17)	O52T—V14—O39B	101.8 (3)
O13B—V1—V5	82.68 (17)	O40B—V14—O39B	154.3 (2)
O7B—V1—V5	127.61 (16)	O45B—V14—O39B	83.7 (2)
O1D—V1—V5	50.29 (12)	O48B—V14—O39B	82.0 (2)
O21T—V1—Pt1	136.0 (2)	O52T—V14—O29D	176.2 (3)
O12B—V1—Pt1	121.12 (17)	O40B—V14—O29D	79.9 (2)
O16B—V1—Pt1	77.65 (16)	O45B—V14—O29D	76.9 (2)
O13B—V1—Pt1	77.00 (16)	O48B—V14—O29D	75.3 (2)
O7B—V1—Pt1	38.78 (15)	O39B—V14—O29D	74.43 (19)
O1D—V1—Pt1	38.63 (12)	O52T—V14—V10	135.7 (2)
V5—V1—Pt1	88.92 (4)	O40B—V14—V10	31.87 (17)
O22T—V2—O9B	104.1 (3)	O45B—V14—V10	82.04 (17)
O22T—V2—O14B	103.9 (3)	O48B—V14—V10	84.85 (17)
O9B—V2—O14B	93.1 (2)	O39B—V14—V10	122.54 (16)
O22T—V2—O15B	104.7 (3)	O29D—V14—V10	48.14 (12)
O9B—V2—O15B	89.2 (2)	O53T—V15—O45B	102.3 (3)
O14B—V2—O15B	149.8 (2)	O53T—V15—O41B	103.9 (3)
O22T—V2—O8B	97.8 (3)	O45B—V15—O41B	92.5 (2)
O9B—V2—O8B	158.1 (2)	O53T—V15—O33C	102.1 (3)
O14B—V2—O8B	83.4 (2)	O45B—V15—O33C	90.3 (2)
O15B—V2—O8B	83.3 (2)	O41B—V15—O33C	152.6 (2)
O22T—V2—O2D	175.0 (3)	O53T—V15—O31C	100.0 (3)
O9B—V2—O2D	80.9 (2)	O45B—V15—O31C	156.3 (2)
O14B—V2—O2D	75.8 (2)	O41B—V15—O31C	89.7 (2)
O15B—V2—O2D	74.9 (2)	O33C—V15—O31C	77.4 (2)
O8B—V2—O2D	77.25 (19)	O53T—V15—O29D	177.4 (2)
O22T—V2—V3	135.9 (2)	O45B—V15—O29D	79.6 (2)
O9B—V2—V3	31.83 (16)	O41B—V15—O29D	77.7 (2)
O14B—V2—V3	84.70 (17)	O33C—V15—O29D	76.0 (2)
O15B—V2—V3	81.58 (17)	O31C—V15—O29D	77.85 (19)
O8B—V2—V3	126.31 (15)	O53T—V15—V16	91.9 (2)
O2D—V2—V3	49.08 (13)	O45B—V15—V16	130.58 (17)
O22T—V2—Pt1	136.4 (2)	O41B—V15—V16	129.82 (17)
O9B—V2—Pt1	119.47 (17)	O33C—V15—V16	40.32 (15)
O14B—V2—Pt1	76.32 (16)	O31C—V15—V16	40.33 (15)
O15B—V2—Pt1	76.36 (16)	O29D—V15—V16	85.47 (13)
O8B—V2—Pt1	38.68 (14)	O53T—V15—Pt2	139.1 (2)
O2D—V2—Pt1	38.58 (12)	O45B—V15—Pt2	118.55 (17)
V3—V2—Pt1	87.65 (4)	O41B—V15—Pt2	78.08 (16)
O23T—V3—O9B	103.8 (3)	O33C—V15—Pt2	76.71 (15)
O23T—V3—O19B	105.1 (3)	O31C—V15—Pt2	39.20 (15)
O9B—V3—O19B	92.0 (2)	O29D—V15—Pt2	38.98 (13)
O23T—V3—O18B	102.5 (3)	V16—V15—Pt2	60.33 (3)
O9B—V3—O18B	89.7 (2)	O54T—V16—O42B	104.9 (3)
O19B—V3—O18B	151.1 (2)	O54T—V16—O46B	103.4 (3)
O23T—V3—O10B	101.3 (3)	O42B—V16—O46B	94.8 (2)
O9B—V3—O10B	154.8 (2)	O54T—V16—O31C	98.2 (2)
O19B—V3—O10B	83.7 (2)	O42B—V16—O31C	91.3 (2)
O18B—V3—O10B	82.6 (2)	O46B—V16—O31C	155.2 (2)
O23T—V3—O2D	174.7 (3)	O54T—V16—O33C	99.6 (3)
O9B—V3—O2D	81.0 (2)	O42B—V16—O33C	153.6 (2)
O19B—V3—O2D	76.7 (2)	O46B—V16—O33C	88.8 (2)
O18B—V3—O2D	75.1 (2)	O31C—V16—O33C	75.5 (2)
O10B—V3—O2D	73.89 (19)	O54T—V16—O30D	173.3 (2)
O23T—V3—V4	131.2 (2)	O42B—V16—O30D	80.8 (2)
O9B—V3—V4	124.91 (17)	O46B—V16—O30D	79.4 (2)
O19B—V3—V4	77.94 (17)	O31C—V16—O30D	77.92 (19)
O18B—V3—V4	77.53 (16)	O33C—V16—O30D	74.2 (2)
O10B—V3—V4	29.94 (15)	O54T—V16—V15	89.4 (2)
O2D—V3—V4	43.96 (12)	O42B—V16—V15	130.56 (17)
O23T—V3—V2	135.5 (2)	O46B—V16—V15	128.07 (18)
O9B—V3—V2	31.72 (17)	O31C—V16—V15	39.37 (14)
O19B—V3—V2	84.66 (17)	O33C—V16—V15	39.29 (14)
O18B—V3—V2	81.72 (17)	O30D—V16—V15	84.14 (13)
O10B—V3—V2	123.10 (16)	O54T—V16—Pt2	137.5 (2)
O2D—V3—V2	49.23 (12)	O42B—V16—Pt2	79.19 (17)
V4—V3—V2	93.19 (5)	O46B—V16—Pt2	118.62 (18)
O23T—V3—Na9	60.4 (2)	O31C—V16—Pt2	39.41 (14)
O9B—V3—Na9	76.93 (19)	O33C—V16—Pt2	76.17 (15)
O19B—V3—Na9	157.87 (19)	O30D—V16—Pt2	39.23 (12)
O18B—V3—Na9	49.40 (18)	V15—V16—Pt2	60.30 (3)
O10B—V3—Na9	114.31 (18)	O56T—V17—O47B	102.2 (3)
O2D—V3—Na9	119.39 (16)	O56T—V17—O43B	104.6 (3)
V4—V3—Na9	124.12 (10)	O47B—V17—O43B	95.4 (2)
V2—V3—Na9	94.90 (10)	O56T—V17—O32C	98.3 (3)
O10B—V4—O11B	108.3 (3)	O47B—V17—O32C	156.0 (2)
O10B—V4—O6C	98.3 (2)	O43B—V17—O32C	91.3 (2)
O11B—V4—O6C	97.8 (2)	O56T—V17—O34C	99.4 (3)
O10B—V4—O5C	98.0 (2)	O47B—V17—O34C	88.9 (2)
O11B—V4—O5C	98.3 (2)	O43B—V17—O34C	154.1 (2)
O6C—V4—O5C	152.2 (2)	O32C—V17—O34C	75.5 (2)
O10B—V4—O2D	87.7 (2)	O56T—V17—O30D	173.4 (3)
O11B—V4—O2D	163.9 (2)	O47B—V17—O30D	80.1 (2)
O6C—V4—O2D	79.0 (2)	O43B—V17—O30D	81.3 (2)
O5C—V4—O2D	79.2 (2)	O32C—V17—O30D	78.16 (19)
O10B—V4—O1D	163.8 (2)	O34C—V17—O30D	74.30 (19)
O11B—V4—O1D	87.8 (2)	O56T—V17—V18	89.8 (2)
O6C—V4—O1D	78.9 (2)	O47B—V17—V18	126.52 (18)
O5C—V4—O1D	79.2 (2)	O43B—V17—V18	131.95 (17)
O2D—V4—O1D	76.10 (19)	O32C—V17—V18	40.86 (15)
O10B—V4—V3	37.78 (18)	O34C—V17—V18	37.60 (14)
O11B—V4—V3	146.1 (2)	O30D—V17—V18	83.91 (13)
O6C—V4—V3	88.88 (15)	O56T—V17—Pt2	137.7 (2)
O5C—V4—V3	90.29 (15)	O47B—V17—Pt2	119.53 (17)
O2D—V4—V3	49.98 (14)	O43B—V17—Pt2	79.64 (17)
O1D—V4—V3	126.07 (14)	O32C—V17—Pt2	39.42 (14)
O10B—V4—Pt1	125.93 (18)	O34C—V17—Pt2	76.02 (15)
O11B—V4—Pt1	125.73 (19)	O30D—V17—Pt2	39.42 (12)
O6C—V4—Pt1	75.88 (15)	V18—V17—Pt2	60.72 (3)
O5C—V4—Pt1	76.31 (15)	O55T—V18—O48B	105.3 (3)
O2D—V4—Pt1	38.18 (14)	O55T—V18—O44B	101.9 (3)
O1D—V4—Pt1	37.92 (13)	O48B—V18—O44B	92.8 (2)
V3—V4—Pt1	88.15 (4)	O55T—V18—O34C	103.1 (3)
O24T—V5—O17B	104.5 (3)	O48B—V18—O34C	92.5 (2)
O24T—V5—O12B	105.9 (3)	O44B—V18—O34C	152.0 (2)
O17B—V5—O12B	92.4 (2)	O55T—V18—O32C	96.5 (3)
O24T—V5—O20B	103.6 (3)	O48B—V18—O32C	157.5 (2)
O17B—V5—O20B	150.0 (2)	O44B—V18—O32C	88.1 (2)
O12B—V5—O20B	89.8 (2)	O34C—V18—O32C	76.9 (2)
O24T—V5—O11B	100.5 (3)	O55T—V18—O29D	173.5 (3)
O17B—V5—O11B	83.9 (2)	O48B—V18—O29D	81.1 (2)
O12B—V5—O11B	153.4 (2)	O44B—V18—O29D	76.8 (2)
O20B—V5—O11B	81.0 (2)	O34C—V18—O29D	76.9 (2)
O24T—V5—O1D	173.9 (3)	O32C—V18—O29D	77.16 (19)
O17B—V5—O1D	76.0 (2)	O55T—V18—V17	90.8 (2)
O12B—V5—O1D	80.0 (2)	O48B—V18—V17	132.26 (18)
O20B—V5—O1D	74.98 (19)	O44B—V18—V17	128.03 (18)
O11B—V5—O1D	73.46 (19)	O34C—V18—V17	39.74 (15)
O24T—V5—V1	137.9 (2)	O32C—V18—V17	40.16 (14)
O17B—V5—V1	83.64 (17)	O29D—V18—V17	85.18 (13)
O12B—V5—V1	31.96 (16)	O55T—V18—Pt2	135.1 (2)
O20B—V5—V1	82.54 (17)	O48B—V18—Pt2	119.60 (18)
O11B—V5—V1	121.53 (15)	O44B—V18—Pt2	77.19 (17)
O1D—V5—V1	48.09 (12)	O34C—V18—Pt2	76.33 (16)
O25T—V6—O13B	104.8 (3)	O32C—V18—Pt2	38.89 (14)
O25T—V6—O17B	102.3 (3)	O29D—V18—Pt2	38.49 (13)
O13B—V6—O17B	94.0 (2)	V17—V18—Pt2	59.72 (3)
O25T—V6—O3C	98.7 (2)	Pt2—O29D—V13	98.2 (2)
O13B—V6—O3C	91.9 (2)	Pt2—O29D—V18	96.0 (2)
O17B—V6—O3C	155.9 (2)	V13—O29D—V18	90.60 (19)
O25T—V6—O5C	100.9 (3)	Pt2—O29D—V15	93.6 (2)
O13B—V6—O5C	153.2 (2)	V13—O29D—V15	90.63 (19)
O17B—V6—O5C	88.0 (2)	V18—O29D—V15	170.1 (3)
O3C—V6—O5C	76.5 (2)	Pt2—O29D—V10	93.6 (2)
O25T—V6—O1D	175.2 (2)	V13—O29D—V10	167.9 (3)
O13B—V6—O1D	79.2 (2)	V18—O29D—V10	91.21 (18)
O17B—V6—O1D	79.8 (2)	V15—O29D—V10	85.57 (17)
O3C—V6—O1D	78.45 (19)	Pt2—O29D—V14	175.8 (3)
O5C—V6—O1D	74.86 (19)	V13—O29D—V14	85.67 (18)
O25T—V6—Pt1	138.3 (2)	V18—O29D—V14	85.54 (18)
O13B—V6—Pt1	79.13 (17)	V15—O29D—V14	84.72 (17)
O17B—V6—Pt1	119.03 (16)	V10—O29D—V14	82.54 (16)
O3C—V6—Pt1	39.77 (14)	Pt2—O30D—V13	100.4 (2)
O5C—V6—Pt1	76.55 (15)	Pt2—O30D—V16	94.5 (2)
O1D—V6—Pt1	39.24 (12)	V13—O30D—V16	93.9 (2)
O25T—V6—V7	91.0 (2)	Pt2—O30D—V17	94.0 (2)
O13B—V6—V7	131.65 (17)	V13—O30D—V17	93.8 (2)
O17B—V6—V7	127.32 (17)	V16—O30D—V17	167.3 (2)
O3C—V6—V7	40.04 (15)	Pt2—O30D—V11	91.9 (2)
O5C—V6—V7	39.33 (14)	V13—O30D—V11	167.7 (2)
O1D—V6—V7	84.38 (13)	V16—O30D—V11	85.03 (17)
Pt1—V6—V7	60.35 (3)	V17—O30D—V11	85.29 (17)
O26T—V7—O18B	102.9 (3)	Pt2—O30D—V12	172.9 (3)
O26T—V7—O14B	104.7 (3)	V13—O30D—V12	86.73 (18)
O18B—V7—O14B	94.4 (2)	V16—O30D—V12	85.41 (17)
O26T—V7—O5C	100.3 (2)	V17—O30D—V12	84.97 (16)
O18B—V7—O5C	90.7 (2)	V11—O30D—V12	80.97 (15)
O14B—V7—O5C	152.6 (2)	V15—O31C—Pt2	102.2 (2)
O26T—V7—O3C	98.2 (3)	V15—O31C—V16	100.3 (2)
O18B—V7—O3C	156.8 (2)	Pt2—O31C—V16	100.8 (2)
O14B—V7—O3C	89.3 (2)	Pt2—O32C—V17	100.8 (2)
O5C—V7—O3C	76.1 (2)	Pt2—O32C—V18	101.1 (2)
O26T—V7—O2D	174.8 (2)	V17—O32C—V18	99.0 (2)
O18B—V7—O2D	80.0 (2)	V13—O33C—V15	110.5 (3)
O14B—V7—O2D	79.2 (2)	V13—O33C—V16	108.7 (2)
O5C—V7—O2D	75.24 (19)	V15—O33C—V16	100.4 (2)
O3C—V7—O2D	78.27 (19)	V13—O34C—V18	110.3 (2)
O26T—V7—V6	90.4 (2)	V13—O34C—V17	108.4 (2)
O18B—V7—V6	130.40 (17)	V18—O34C—V17	102.7 (2)
O14B—V7—V6	128.33 (18)	Pt2—O35B—V10	101.2 (2)
O5C—V7—V6	39.71 (15)	Pt2—O35B—H35	114 (6)
O3C—V7—V6	39.24 (14)	V10—O35B—H35	112 (6)
O2D—V7—V6	84.51 (13)	Pt2—O36B—V11	102.6 (2)
O26T—V7—Pt1	137.7 (2)	Pt2—O36B—H36	115 (6)
O18B—V7—Pt1	119.17 (18)	V11—O36B—H36	117 (6)
O14B—V7—Pt1	77.68 (16)	V12—O37B—V11	117.1 (3)
O5C—V7—Pt1	76.32 (14)	V13—O38B—V12	111.6 (3)
O3C—V7—Pt1	39.57 (14)	V13—O39B—V14	113.6 (3)
O2D—V7—Pt1	39.22 (13)	V14—O40B—V10	116.7 (3)
V6—V7—Pt1	59.79 (3)	V10—O41B—V15	118.0 (3)
O27T—V8—O15B	104.2 (3)	V16—O42B—V11	117.2 (3)
O27T—V8—O19B	102.8 (3)	V17—O43B—V11	116.0 (3)
O15B—V8—O19B	94.1 (2)	V18—O44B—V10	116.6 (3)
O27T—V8—O4C	98.2 (3)	V18—O44B—H44	129 (6)
O15B—V8—O4C	92.4 (2)	V10—O44B—H44	114 (6)
O19B—V8—O4C	155.8 (2)	V15—O45B—V14	118.3 (3)
O27T—V8—O6C	101.4 (3)	V16—O46B—V12	118.4 (3)
O15B—V8—O6C	153.1 (2)	V17—O47B—V12	117.7 (3)
O19B—V8—O6C	88.1 (2)	V18—O48B—V14	117.5 (3)
O4C—V8—O6C	75.9 (2)	V11—O50T—O15B	118.8 (3)
O27T—V8—O2D	175.0 (2)	O29W—Na1—O27W	100.4 (3)
O15B—V8—O2D	79.8 (2)	O29W—Na1—O12W	96.9 (2)
O19B—V8—O2D	79.7 (2)	O27W—Na1—O12W	93.8 (3)
O4C—V8—O2D	78.5 (2)	O29W—Na1—O1W	165.5 (3)
O6C—V8—O2D	74.26 (19)	O27W—Na1—O1W	86.1 (3)
O27T—V8—Pt1	137.5 (2)	O12W—Na1—O1W	95.6 (3)
O15B—V8—Pt1	79.50 (18)	O29W—Na1—O28W	83.7 (3)
O19B—V8—Pt1	119.27 (18)	O27W—Na1—O28W	85.7 (3)
O4C—V8—Pt1	39.58 (14)	O12W—Na1—O28W	179.3 (3)
O6C—V8—Pt1	76.10 (15)	O1W—Na1—O28W	83.9 (3)
O2D—V8—Pt1	39.60 (13)	O29W—Na1—O2W	83.8 (2)
O28T—V9—O20B	103.0 (3)	O27W—Na1—O2W	147.5 (3)
O28T—V9—O16B	104.0 (3)	O12W—Na1—O2W	117.8 (3)
O20B—V9—O16B	94.6 (2)	O1W—Na1—O2W	83.9 (3)
O28T—V9—O6C	100.5 (3)	O28W—Na1—O2W	62.6 (3)
O20B—V9—O6C	91.2 (2)	O3W—Na2—O30W	97.7 (3)
O16B—V9—O6C	152.8 (2)	O3W—Na2—O31W	165.1 (3)
O28T—V9—O4C	98.4 (2)	O30W—Na2—O31W	90.3 (2)
O20B—V9—O4C	156.8 (2)	O3W—Na2—O13W	85.3 (3)
O16B—V9—O4C	88.8 (2)	O30W—Na2—O13W	175.3 (3)
O6C—V9—O4C	76.0 (2)	O31W—Na2—O13W	86.0 (2)
O28T—V9—O1D	174.6 (2)	O3W—Na2—O6W	84.5 (3)
O20B—V9—O1D	80.9 (2)	O30W—Na2—O6W	90.2 (2)
O16B—V9—O1D	79.2 (2)	O31W—Na2—O6W	82.9 (2)
O6C—V9—O1D	75.5 (2)	O13W—Na2—O6W	86.4 (2)
O4C—V9—O1D	77.26 (19)	O3W—Na2—O12W	101.4 (3)
O28T—V9—Pt1	137.3 (2)	O30W—Na2—O12W	90.3 (2)
O20B—V9—Pt1	119.51 (17)	O31W—Na2—O12W	91.2 (2)
O16B—V9—Pt1	78.31 (16)	O13W—Na2—O12W	92.8 (2)
O6C—V9—Pt1	75.68 (15)	O6W—Na2—O12W	174.0 (2)
O4C—V9—Pt1	38.97 (14)	O5W—Na3—O13W	93.8 (3)
O1D—V9—Pt1	38.65 (12)	O5W—Na3—O4W	107.2 (3)
Pt1—O1D—V4	99.4 (2)	O13W—Na3—O4W	101.7 (2)
Pt1—O1D—V9	95.6 (2)	O5W—Na3—O14W	173.2 (3)
V4—O1D—V9	91.78 (19)	O13W—Na3—O14W	83.7 (2)
Pt1—O1D—V6	93.9 (2)	O4W—Na3—O14W	79.6 (2)
V4—O1D—V6	93.01 (19)	O5W—Na3—O17W	93.2 (3)
V9—O1D—V6	168.5 (2)	O13W—Na3—O17W	167.2 (3)
Pt1—O1D—V1	93.5 (2)	O4W—Na3—O17W	86.4 (2)
V4—O1D—V1	167.1 (2)	O14W—Na3—O17W	88.2 (2)
V9—O1D—V1	86.79 (17)	O5W—Na3—O6W	93.6 (2)
V6—O1D—V1	86.21 (17)	O13W—Na3—O6W	86.1 (2)
Pt1—O1D—V5	175.0 (3)	O4W—Na3—O6W	157.1 (3)
V4—O1D—V5	85.52 (18)	O14W—Na3—O6W	80.0 (2)
V9—O1D—V5	85.31 (17)	O17W—Na3—O6W	82.8 (2)
V6—O1D—V5	84.66 (17)	O8W—Na4—O22W	95.2 (2)
V1—O1D—V5	81.62 (16)	O8W—Na4—O20W	96.8 (3)
Pt1—O2D—V4	99.5 (2)	O22W—Na4—O20W	87.9 (2)
Pt1—O2D—V7	94.8 (2)	O8W—Na4—O33W	106.4 (3)
V4—O2D—V7	92.8 (2)	O22W—Na4—O33W	98.9 (2)
Pt1—O2D—V8	93.6 (2)	O20W—Na4—O33W	155.0 (2)
V4—O2D—V8	93.51 (19)	O8W—Na4—O18W	91.8 (2)
V7—O2D—V8	168.6 (2)	O22W—Na4—O18W	169.3 (2)
Pt1—O2D—V3	174.3 (3)	O20W—Na4—O18W	83.3 (2)
V4—O2D—V3	86.07 (18)	O33W—Na4—O18W	86.8 (2)
V7—O2D—V3	85.94 (17)	O8W—Na4—O19W	176.3 (3)
V8—O2D—V3	85.00 (18)	O22W—Na4—O19W	83.1 (2)
Pt1—O2D—V2	92.8 (2)	O20W—Na4—O19W	79.8 (2)
V4—O2D—V2	167.7 (3)	O33W—Na4—O19W	77.2 (2)
V7—O2D—V2	85.56 (17)	O18W—Na4—O19W	89.4 (2)
V8—O2D—V2	86.25 (18)	O9W—Na5—O10W	179.4 (3)
V3—O2D—V2	81.69 (16)	O9W—Na5—O22W	88.5 (2)
V6—O3C—Pt1	101.0 (2)	O10W—Na5—O22W	92.2 (3)
V6—O3C—V7	100.7 (2)	O9W—Na5—O20W	84.9 (2)
Pt1—O3C—V7	100.6 (2)	O10W—Na5—O20W	95.0 (3)
Pt1—O4C—V8	100.5 (2)	O22W—Na5—O20W	88.2 (2)
Pt1—O4C—V9	101.0 (2)	O9W—Na5—O23W	88.6 (2)
V8—O4C—V9	99.9 (2)	O10W—Na5—O23W	91.5 (3)
V4—O5C—V7	108.9 (2)	O22W—Na5—O23W	92.0 (2)
V4—O5C—V6	109.7 (2)	O20W—Na5—O23W	173.5 (2)
V7—O5C—V6	101.0 (2)	O9W—Na5—O21W	85.8 (3)
V4—O6C—V9	109.3 (2)	O10W—Na5—O21W	93.6 (3)
V4—O6C—V8	109.8 (2)	O22W—Na5—O21W	174.3 (2)
V9—O6C—V8	102.2 (2)	O20W—Na5—O21W	91.7 (2)
Pt1—O7B—V1	101.4 (2)	O23W—Na5—O21W	87.5 (2)
Pt1—O7B—H7	115 (6)	O23W—Na6—O11W	166.4 (3)
V1—O7B—H7	108 (6)	O23W—Na6—O25W	91.9 (2)
Pt1—O8B—V2	101.5 (2)	O11W—Na6—O25W	87.3 (3)
Pt1—O8B—H8	100 (6)	O23W—Na6—O21W	88.7 (2)
V2—O8B—H8	128 (6)	O11W—Na6—O21W	94.2 (2)
V2—O9B—V3	116.5 (3)	O25W—Na6—O21W	171.0 (3)
V4—O10B—V3	112.3 (3)	O23W—Na6—O24W	86.0 (2)
V4—O11B—V5	113.2 (3)	O11W—Na6—O24W	81.0 (3)
V1—O12B—V5	115.8 (3)	O25W—Na6—O24W	104.2 (3)
V6—O13B—V1	117.5 (3)	O21W—Na6—O24W	84.8 (3)
V2—O14B—V7	117.6 (3)	O23W—Na6—O34W	99.5 (3)
V8—O15B—V2	118.1 (3)	O11W—Na6—O34W	93.9 (3)
V8—O15B—O50T	106.1 (2)	O25W—Na6—O34W	83.6 (3)
V2—O15B—O50T	129.2 (3)	O21W—Na6—O34W	87.5 (3)
V9—O16B—V1	115.7 (3)	O24W—Na6—O34W	170.4 (3)
V5—O17B—V6	118.3 (3)	O7W—Na7—O14W	78.9 (2)
V7—O18B—V3	118.2 (3)	O7W—Na7—O15W	170.2 (3)
V3—O19B—V8	117.3 (3)	O14W—Na7—O15W	94.0 (2)
V9—O20B—V5	117.9 (3)	O7W—Na7—O16W	106.5 (2)
O29D—Pt2—O30D	85.5 (2)	O14W—Na7—O16W	94.8 (2)
O29D—Pt2—O32C	85.2 (2)	O15W—Na7—O16W	80.7 (2)
O30D—Pt2—O32C	85.7 (2)	O7W—Na7—O17W	92.0 (2)
O29D—Pt2—O35B	87.8 (2)	O14W—Na7—O17W	87.8 (2)
O30D—Pt2—O35B	173.2 (2)	O15W—Na7—O17W	80.9 (2)
O32C—Pt2—O35B	94.9 (2)	O16W—Na7—O17W	161.5 (3)
O29D—Pt2—O31C	85.7 (2)	O7W—Na7—O22T^ii^	86.9 (2)
O30D—Pt2—O31C	85.2 (2)	O14W—Na7—O22T^ii^	165.3 (2)
O32C—Pt2—O31C	167.6 (2)	O15W—Na7—O22T^ii^	100.5 (2)
O35B—Pt2—O31C	93.2 (2)	O16W—Na7—O22T^ii^	85.5 (2)
O29D—Pt2—O36B	173.7 (2)	O17W—Na7—O22T^ii^	96.5 (2)
O30D—Pt2—O36B	88.2 (2)	O19W—Na8—O49T^i^	166.9 (2)
O32C—Pt2—O36B	95.0 (2)	O19W—Na8—O16W^i^	94.8 (2)
O35B—Pt2—O36B	98.5 (2)	O49T^i^—Na8—O16W^i^	96.9 (2)
O31C—Pt2—O36B	93.1 (2)	O19W—Na8—O32W	84.5 (2)
O29D—Pt2—V17	90.02 (14)	O49T^i^—Na8—O32W	85.0 (2)
O30D—Pt2—V17	46.60 (15)	O16W^i^—Na8—O32W	169.4 (3)
O32C—Pt2—V17	39.82 (14)	O19W—Na8—O15W^i^	92.8 (2)
O35B—Pt2—V17	134.64 (15)	O49T^i^—Na8—O15W^i^	83.3 (2)
O31C—Pt2—V17	131.80 (14)	O16W^i^—Na8—O15W^i^	80.5 (2)
O36B—Pt2—V17	86.12 (15)	O32W—Na8—O15W^i^	110.1 (3)
O29D—Pt2—V15	47.45 (15)	O19W—Na8—O18W	90.8 (2)
O30D—Pt2—V15	88.06 (15)	O49T^i^—Na8—O18W	96.8 (2)
O32C—Pt2—V15	132.61 (15)	O16W^i^—Na8—O18W	81.1 (2)
O35B—Pt2—V15	86.57 (15)	O32W—Na8—O18W	88.3 (3)
O31C—Pt2—V15	38.63 (14)	O15W^i^—Na8—O18W	161.5 (3)
O36B—Pt2—V15	131.77 (15)	O24W^iii^—Na9—O26W	114.8 (4)
V17—Pt2—V15	123.17 (3)	O24W^iii^—Na9—O26W^iv^	88.6 (3)
O29D—Pt2—V16	90.58 (15)	O26W—Na9—O26W^iv^	90.2 (3)
O30D—Pt2—V16	46.23 (15)	O24W^iii^—Na9—O25W^v^	158.6 (3)
O32C—Pt2—V16	131.88 (14)	O26W—Na9—O25W^v^	86.1 (3)
O35B—Pt2—V16	132.89 (15)	O26W^iv^—Na9—O25W^v^	86.9 (3)
O31C—Pt2—V16	39.78 (14)	O24W^iii^—Na9—O18B	77.0 (3)
O36B—Pt2—V16	84.62 (15)	O26W—Na9—O18B	94.9 (3)
V17—Pt2—V16	92.42 (3)	O26W^iv^—Na9—O18B	165.5 (3)
V15—Pt2—V16	59.37 (3)	O25W^v^—Na9—O18B	107.0 (3)
O29D—Pt2—V18	45.50 (15)	O24W^iii^—Na9—O23T	93.2 (3)
O30D—Pt2—V18	87.66 (15)	O26W—Na9—O23T	138.4 (3)
O32C—Pt2—V18	39.97 (15)	O26W^iv^—Na9—O23T	122.2 (3)
O35B—Pt2—V18	88.41 (15)	O25W^v^—Na9—O23T	71.8 (2)
O31C—Pt2—V18	131.12 (14)	O18B—Na9—O23T	60.72 (19)
O36B—Pt2—V18	134.92 (15)	H1A—O1W—H1B	100 (4)
V17—Pt2—V18	59.56 (3)	H2A—O2W—H2B	101 (4)
V15—Pt2—V18	92.92 (3)	H3A—O3W—H3B	107 (5)
V16—Pt2—V18	121.99 (4)	H4A—O4W—H4B	114 (5)
O29D—Pt2—V10	47.89 (14)	H5A—O5W—H5B	106 (5)
O30D—Pt2—V10	133.33 (15)	H6A—O6W—H6B	110 (5)
O32C—Pt2—V10	91.75 (15)	H7A—O7W—H7B	116 (5)
O35B—Pt2—V10	39.90 (15)	H8A—O8W—H8B	110 (5)
O31C—Pt2—V10	88.43 (14)	H9A—O9W—H9B	111 (5)
O36B—Pt2—V10	138.32 (15)	H10A—O10W—H10B	103 (5)
V17—Pt2—V10	122.46 (4)	H11A—O11W—H11B	104 (4)
V15—Pt2—V10	60.42 (3)	H12A—O12W—H12B	107.5
V16—Pt2—V10	119.75 (4)	H13A—O13W—H13B	110.2
V18—Pt2—V10	62.92 (3)	H14A—O14W—H14B	110.6
O29D—Pt2—V11	134.82 (15)	H15A—O15W—H15B	109.6
O30D—Pt2—V11	49.32 (15)	H16A—O16W—H16B	109.6
O32C—Pt2—V11	90.33 (15)	H17A—O17W—H17B	110.7
O35B—Pt2—V11	137.40 (15)	H18A—O18W—H18B	111.0
O31C—Pt2—V11	90.06 (14)	H19A—O19W—H19B	110.8
O36B—Pt2—V11	38.92 (15)	H20A—O20W—H20B	110.7
V17—Pt2—V11	60.53 (4)	H21A—O21W—H21B	110.7
V15—Pt2—V11	119.55 (4)	H22A—O22W—H22B	110.6
V16—Pt2—V11	60.19 (3)	H23A—O23W—H23B	110.6
V18—Pt2—V11	120.09 (3)	H24A—O24W—H24B	107.6
V10—Pt2—V11	176.76 (4)	H25A—O25W—H25B	106.3
O49T—V10—O41B	105.2 (3)	H26A—O26W—H26B	110.9
O49T—V10—O40B	104.0 (3)	H28A—O28W—H28B	109.5
O41B—V10—O40B	93.0 (2)	H29A—O29W—H29B	109.5
O49T—V10—O44B	102.9 (3)	H30A—O30W—H30B	109.5
O41B—V10—O44B	150.8 (2)	H31A—O31W—H31B	109.5
O40B—V10—O44B	87.8 (2)	H32A—O32W—H32B	109.5
O49T—V10—O35B	97.7 (3)	H33A—O33W—H33B	109.5
O41B—V10—O35B	85.0 (2)	H34A—O34W—H34B	109.5

Symmetry codes: (i) *x*−1, *y*, *z*; (ii) *x*+1, *y*, *z*; (iii) −*x*+1, −*y*+2, −*z*+1; (iv) −*x*, −*y*+1, −*z*+1; (v) *x*−1, *y*−1, *z*.

### Hydrogen-bond geometry (Å, º)

**Table:**

*D*—H···*A*	*D*—H	H···*A*	*D*···*A*	*D*—H···*A*
O7*B*—H7···O32*C*	0.85 (3)	1.79 (3)	2.627 (7)	171 (8)
O8*B*—H8···O43*B*	0.86 (3)	1.89 (3)	2.737 (7)	169 (8)
O35*B*—H35···O16*B*	0.85 (3)	1.86 (4)	2.685 (7)	164 (9)
O36*B*—H36···O4*C*	0.84 (3)	1.79 (3)	2.628 (7)	174 (8)
O44*B*—H44···O21*T*	0.84 (3)	2.00 (6)	2.724 (8)	144 (8)
O1*W*—H1*B*···O40*B*^v^	0.90 (3)	2.18 (9)	2.845 (9)	130 (9)
O2*W*—H2*B*···O47*B*^vi^	0.88 (3)	1.88 (3)	2.757 (8)	178 (12)
O3*W*—H3*A*···O39*B*^vi^	0.86 (3)	2.01 (4)	2.856 (9)	168 (10)
O3*W*—H3*B*···O33*C*^vii^	0.87 (3)	1.97 (6)	2.795 (8)	159 (12)
O4*W*—H4*A*···O51*T*^ii^	0.84 (3)	2.08 (3)	2.893 (8)	163 (8)
O4*W*—H4*B*···O41*B*	0.84 (3)	1.96 (4)	2.780 (8)	165 (10)
O5*W*—H5*B*···O46*B*^vii^	0.86 (3)	1.94 (5)	2.743 (8)	154 (9)
O6*W*—H6*A*···O7*W*	0.85 (3)	2.03 (3)	2.872 (9)	170 (9)
O6*W*—H6*B*···O48*B*^vi^	0.84 (3)	2.02 (5)	2.810 (8)	158 (9)
O7*W*—H7*A*···O28*T*	0.83 (3)	2.26 (5)	2.951 (8)	141 (8)
O7*W*—H7*B*···O34*W*^vi^	0.84 (3)	2.05 (6)	2.794 (10)	148 (8)
O8*W*—H8*A*···O17*B*^iii^	0.86 (3)	1.93 (5)	2.760 (8)	161 (9)
O8*W*—H8*B*···O23*T*^viii^	0.85 (3)	2.12 (7)	2.872 (9)	148 (10)
O9*W*—H9*A*···O7*B*	0.85 (3)	2.01 (5)	2.826 (8)	161 (9)
O9*W*—H9*B*···O37*W*	0.84 (3)	2.39 (6)	3.073 (11)	138 (8)
O10*W*—H10*A*···O5*C*^iii^	0.88 (3)	1.92 (4)	2.782 (9)	165 (11)
O11*W*—H11*A*···O22*T*^ix^	0.87 (3)	2.23 (5)	3.061 (8)	158 (9)
O12*W*—H12*A*···O42*B*	0.99	1.92	2.861 (8)	159
O12*W*—H12*B*···O53*T*^vii^	0.99	2.00	2.956 (8)	160
O13*W*—H13*A*···O31*C*	0.99	1.84	2.831 (7)	176
O13*W*—H13*B*···O54*T*^vii^	0.99	1.93	2.905 (8)	169
O14*W*—H14*A*···O35*B*	0.99	1.96	2.806 (8)	142
O14*W*—H14*B*···O31*W*	0.99	2.03	2.928 (8)	151
O15*W*—H15*A*···O4*W*	0.99	1.92	2.819 (9)	149
O16*W*—H16*A*···O20*B*	0.99	2.51	3.054 (7)	115
O16*W*—H16*B*···O33*W*^ii^	0.99	1.88	2.764 (9)	147
O17*W*—H17*A*···O37*B*^ii^	0.99	2.06	3.013 (8)	162
O17*W*—H17*A*···O50*T*^ii^	0.99	2.60	3.205 (8)	119
O18*W*—H18*A*···O39*W*^viii^	0.99	1.97	2.942 (10)	167
O18*W*—H18*B*···O12*B*^i^	0.99	2.01	2.977 (8)	165
O19*W*—H19*A*···O9*W*	0.99	2.05	2.949 (9)	149
O19*W*—H19*B*···O8*B*	0.99	1.99	2.867 (8)	146
O20*W*—H20*A*···O32*W*	0.99	1.94	2.857 (9)	152
O20*W*—H20*B*···O19*B*^viii^	0.99	1.81	2.753 (8)	157
O21*W*—H21*A*···O37*W*	0.99	1.93	2.836 (10)	151
O21*W*—H21*B*···O6*C*^viii^	0.99	1.90	2.869 (8)	164
O22*W*—H22*A*···O25*T*^iii^	0.99	1.94	2.920 (8)	170
O22*W*—H22*B*···O3*C*	0.99	1.83	2.819 (7)	173
O23*W*—H23*A*···O26*T*^iii^	0.99	2.04	2.993 (8)	160
O23*W*—H23*B*···O13*B*	0.99	1.87	2.840 (8)	167
O24*W*—H24*A*···O11*B*^viii^	0.99	1.97	2.887 (9)	152
O24*W*—H24*B*···O10*W*	0.99	2.15	3.082 (13)	157
O25*W*—H25*A*···O9*B*^ix^	0.99	2.07	2.874 (9)	137
O25*W*—H25*B*···O35*W*	0.99	1.93	2.880 (10)	160
O26*W*—H26*A*···O11*W*^v^	0.99	1.88	2.848 (11)	165
O26*W*—H26*B*···O38*W*	0.99	1.86	2.811 (12)	161
O27*W*—H27*A*···O36*W*	0.98	2.11	2.881 (10)	135
O27*W*—H27*B*···O29*W*^x^	0.98	2.09	2.891 (10)	138
O28*W*—H28*A*···O27*W*^x^	0.98	1.78	2.692 (11)	153
O28*W*—H28*B*···O40*B*^v^	0.98	2.45	2.994 (9)	115
O29*W*—H29*A*···O38*B*^vi^	0.98	2.12	2.992 (8)	147
O29*W*—H29*B*···O45*B*^vii^	0.98	1.93	2.755 (8)	141
O30*W*—H30*A*···O2*W*	0.98	2.03	2.877 (10)	143
O30*W*—H30*B*···O37*W*^vi^	0.98	1.93	2.893 (10)	167
O31*W*—H31*B*···O36*B*	0.98	2.08	2.812 (8)	130
O32*W*—H32*A*···O40*W*^viii^	0.98	1.96	2.901 (13)	160
O32*W*—H32*B*···O2*W*^viii^	0.98	2.01	2.896 (10)	150
O33*W*—H33*A*···O38*W*	0.98	2.13	2.833 (11)	128
O33*W*—H33*B*···O14*B*	0.98	1.81	2.786 (8)	178
O34*W*—H34*A*···O55*T*	0.98	2.29	3.038 (9)	132
O34*W*—H34*B*···O39*W*^ix^	0.98	2.02	2.979 (12)	166

Symmetry codes: (i) *x*−1, *y*, *z*; (ii) *x*+1, *y*, *z*; (iii) −*x*+1, −*y*+2, −*z*+1; (v) *x*−1, *y*−1, *z*; (vi) *x*, *y*−1, *z*; (vii) −*x*+1, −*y*+1, −*z*; (viii) *x*, *y*+1, *z*; (ix) *x*+1, *y*+1, *z*; (x) −*x*, −*y*, −*z*.
